# Effects of off-line auricular transcutaneous vagus nerve stimulation (taVNS) on a short-term memory task: a pilot study

**DOI:** 10.3389/fnagi.2025.1549167

**Published:** 2025-04-28

**Authors:** Francesco Fisicaro, Klizia Cortese, Rita Bella, Manuela Pennisi, Giuseppe Lanza, Kaoru Yuasa, Yoshikazu Ugawa, Yasuo Terao

**Affiliations:** ^1^Department of Biomedical and Biotechnological Sciences, University of Catania, Catania, Italy; ^2^Department of Medical Physiology, Kyorin University, Shinkawa, Tokyo, Japan; ^3^Department of Educational Sciences, University of Catania, Catania, Italy; ^4^Department of Medical and Surgical Sciences and Advanced Technologies, University of Catania, Catania, Italy; ^5^Department of Surgery and Medical-Surgical Specialties, University of Catania, Catania, Italy; ^6^Clinical Neurophysiology Research Unit, Oasi Research Institute-IRCCS, Troina, Italy; ^7^Department of Human Neurophysiology, School of Medicine, Fukushima Medical University, Fukushima, Japan

**Keywords:** transcutaneous auricular vagus nerve stimulation, pupil size, digit span, short term memory, non-invasive brain stimulation

## Abstract

**Introduction:**

One of the commonly used indices of short-term memory (STM) is the digit span task. Prior studies have proposed pupil dilation as a measure of task engagement and as a promising biomarker of vagal activation. Transcutaneous auricular vagus nerve stimulation (taVNS) is a novel non-invasive brain stimulation technique which might be used to improve cognition and modulate pupil size through its effects on the noradrenergic release in the locus coeruleus. No previous study has investigated the effects of off-line taVNS on a digit span task. With this single-blind, sham-controlled, crossover design trial, we aimed to assess whether taVNS was able to improve the digit span score, as well as to modulate the pupillary response to cognitive load in a sample of 18 elderly Japanese volunteers with no self-reported cognitive impairments.

**Results:**

Subjects were randomized to receive either real or sham taVNS during a digit span task while recording the pupil size, and then switched over to the other treatment group. We found that real stimulation significantly reduced the mean number of errors performed at span length 7, 8, and 9 (–0.83, –0.90, and –0.39, respectively compared to pre-stimulation values, and –0.71, –1.08, and –0.79, respectively, compared to sham stimulation). Additionally, real taVNS stimulation slightly but significantly increased the pupil size at all span lengths during the encoding period of the task, with larger effects for span 7–10 compared to pre-stimulation, and for span 5–10 compared to sham. No effect over the pupil size was found during the recall period.

**Discussion:**

Our results suggest that taVNS might selectively improve the cognitive performance during the encoding phase of the task. Although further studies are needed to better clarify the optimal stimulation parameters, findings from this study could support the use of taVNS as a safe neuromodulation technique to improve cognitive function.

## 1 Introduction

The ability to maintain information for a short period of time, known as short-term memory (STM) is tied to the ability to perform complex cognitive tasks, such as mathematics (Baddeley, 1992; Cowan et al., 2005). One of the commonly used indices of STM is the digit span task, a measure of verbal STM (Cowan et al., 2005). The digit span task requires the encoding and immediate serial recall of a list of numbers presented aurally, and the length of an individual’s span depends on how well the subjects can repeat back the stimuli (Unsworth and Engle, 2007a,b).

Pupil dilation related to physiological arousal is mediated by the simultaneous activation of sympathetic pathways and inhibition of parasympathetic pathways (Beatty and Lucero-Wagoner, 2000), and evidence suggests that task-evoked pupil dilation results from cortical inhibition of the parasympathetic oculomotor nucleus (Steinhauer et al., 2004). During a state of heightened attention, neurons in the locus coeruleus (LC) fire rapidly, supplying noradrenaline to different targets throughout the body, including both the eyes and brain and mediating, respectively, pupil dilation and attention (Laeng et al., 2012).

Task-evoked pupil dilation in experimental settings has been referred to as a peripheral marker of heightened attention, mental effort, or allocation of cognitive control when the task prompts focus or conscious engagement (Beatty, 1982a,b; Richer and Beatty, 1987; Beatty and Lucero-Wagoner, 2000). Several studies have investigated the pupil response to cognitive load, proposing two main patterns: an inverted U-shaped pattern where increasing memory load up to the capacity limit is associated with increasing pupil dilation, while further memory overload is associated with pupil constriction; a simpler, bi-phasic pattern whereby pupil size increases with load and reaches a stable plateau at the capacity limit (Kosachenko et al., 2023). Given the association between pupil size and arousal, the shape of this pattern is indicative of how people respond to cognitive load: while an inverted U-shaped pattern would imply that cognitive effort are released, a prolonged plateau would imply that they are maintained even when task demands exceed the capacity limit (Kosachenko et al., 2023). Pupils also tend to constrict during recall as items are offloaded from STM (Cabestrero et al., 2009). These findings are consistent with the idea that cognitive resources are dedicated in a manner proportionate to the cognitive load and validate pupil dilation as a measure of task engagement (Johnson et al., 2014).

The vagus nerve plays a widespread role maintaining autonomic tone among brain structures and peripheral organs. Release of catecholamines in prefrontal cortex modulates cognitive function to support attentional control and working memory (Arnsten, 1997). Noradrenergic mechanisms can be modulated by vagus nerve stimulation (VNS) (Hulsey et al., 2017) and contribute, at least in part, to therapeutic effects of VNS on treating symptomology associated with a wide spectrum of clinical disorders (Ben-Menachem et al., 2015; Johnson and Wilson, 2018).

Previous studies show evidence that the auricular branch of the vagus nerve activates the nucleus tractus solitarii (NTS) and the LC (Dietrich et al., 2008; Kraus et al., 2013; Frangos et al., 2015, 2015; Yakunina et al., 2017; Liu et al., 2020), and that could be stimulated using transcutaneous auricular VNS (taVNS) (Ben-Menachem et al., 2015).

To date, taVNS is still in its early adoption and there is much debate about the parameters to be used. The current intensity is typically administered above the perceptual threshold and below the pain threshold (Thompson et al., 2021). Several studies have explored the diverse “optimal” parameters for taVNS using different evaluation markers (Geng et al., 2022). In addition to variations in stimulation parameters, the timing of stimulation relative to a given task or measurement can also vary. Stimulation applied before a task is referred to as “off-line,” whereas stimulation applied during a task is referred to as “on-line” (Oldrati et al., 2018). Regarding its possible applications on cognitive function, a meta-analysis showed that both on-line and off-line taVNS may improve cognition, particularly executive function (Ridgewell et al., 2021), although on-line stimulation was not able to improve the digit span scoring (Jacobs et al., 2015).

In recent times, pupil size in particular has gained attention as a promising biomarker of vagal activation (Burger et al., 2020). Previous studies showed that auricular stimulation elicits acute pupillary responses (Urbin et al., 2021). In particular, it has been shown that on-line tVNS can lead to pupil dilation during task-free rest conditions (Sharon et al., 2021). However, the effects of taVNS on pupil size are strictly dependent on technical aspects, such as stimulation parameters and experimental set-up (Capone et al., 2021).

To the best of our knowledge, no previous study has investigated the effect of off-line taVNS on a short-term memory task (i.e., digit span) in healthy volunteers. In this study, we hypothesized that off-line taVNS might be able to improve the subjects’ scores in a digit span task and also modulate the pupillary response, increasing the pupil size during the cognitive load.

## 2 Materials and methods

This study was a single blind, sham controlled, crossover design trial involving healthy elderly volunteers. The aim of the study was to explore the off-line effects of taVNS on a short-term memory task (i.e., digit span). The primary outcome was to score a digit span cognitive task (measured as the total number of errors performed by the subjects), whereas the secondary outcome was to assess the change in pupil diameter during the cognitive task. We expected better scores and a change in pupil size (as an indirect measure of cognitive load) after the real intervention compared to sham stimulation.

Enrolled subjects were randomized with a 1:1 blocked randomization in two intervention sequences: real stimulation followed by sham, or vice versa. The randomization list was created with the RL4 function of the “randomizeBE” package in R, using a block size of 4. The enrolled subjects were then consecutively assigned to the treatment group according to the generated list. Subjects were blinded to the Intervention allocation, while researchers were not blinded due to the technical differences in performing the Real and Sham intervention. To avoid any carry-over effect, subjects underwent a wash-out period of at least 1-week before being switched over to the other intervention.

**FIGURE 1:**
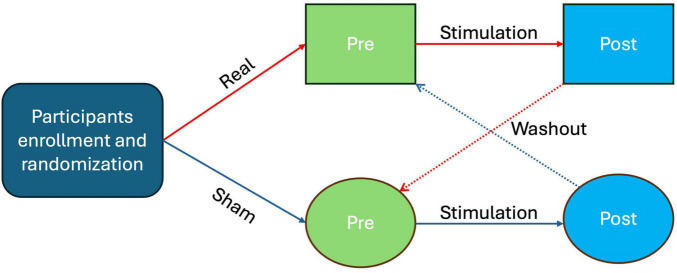
Schematic diagram of the study design.

Each subject performed four sessions of the cognitive task during eye-tracking measurements, as schematized in Figure 1.

### 2.1 Subjects

According to an exploratory analysis we conducted, with a two-sided significance level of 0.05 and a power of 0.8, the estimated required sample size was 16 subjects. Assuming a dropout rate of 10%, 18 subjects were enrolled.

Adult volunteers with no self-reported cognitive impairments were enrolled at the Department of Medical Physiology, Kyorin University, Mitaka, Japan, from January 2023 to July 2023. The cognitive status of the subjects was screened with two well-known neuropsychological scales: Mini-Mental State Examination (MMSE) (Folstein et al., 1975) and Montreal Cognitive Assessment (MoCA) (Nasreddine et al., 2005).

Written informed consent was acquired from all subjects before the enrollment. The study received the local Ethics Committee approval (approval no. 826) and was conducted according to the Declaration of Helsinki and its later amendments.

### 2.2 Experimental task

Subjects were seated in front of a 17-inch monitor screen with a refresh rate of 60 Hz. The monitor was positioned vertically at a viewing distance of 50 cm in front of their eyes. The subjects had to fixate the center of the screen during the task. Tasks were created using the SR Research Experiment Builder software, version 1.5.58. Each session consisted of 63 trials of randomized series of numbers, of different length. Randomization of the sequence prevented any learning effect. The subject was first asked to listen to the numbers presented through a headset at a rate of one per second (encoding phase) and then to repeat the digit sequence after a prompt “*Please repeat the digits*” (recall phase), while keeping the sight fixed at the center of the screen. Pupil size was measured during the encoding and during the recall of the sequences.

The pupil size data was recorded from the dominant eye. The pupil size was measured by a video-based eye tracking system (EyeLink 1000, SR Research, Mississauga, Ontario, Canada) at a sampling rate of 1,000 Hz, and spatial resolution of less than 0.04°. EyeLink reports pupil size as an arbitrary unit, which is based on the number of pixels the pupil consists of, as measured with the eye tracker’s camera. This value was then converted to a diameter in millimeters using a scaling factor according to the EyeLink documentation. The pupil size data were processed using EyeLink Data Viewer software (Data Viewer ver. 1.3.137., SR Research, Mississauga, ON, Canada).

Data on recall accuracy were collected with a recording microphone and later analyzed by an examiner blinded to the intervention.

The total number of errors was defined as the sum of the errors in digit recall (i.e., wrong number recall or failing to recall a number) and in digit placement in the sequence (i.e., a misplaced number in the span sequence).

### 2.3 Stimulation protocol

To reduce potential cardiac side-effects, electrodes were placed only on the left ear (Nemeroff et al., 2006). We applied stimulation through a biphasic electric stimulator (Electronic Stimulator SEN-3401MG and Isolator SS-203JMG, Miyuki Giken Co., Ltd., Tokyo, Japan) and two Ag–AgCl electrodes (5 mm in diameter with a distance between the cathode and anode of about 5 mm), placed in the left external acoustic meatus at the inner side of the tragus for real taVNS and attached to the left ear lobe for sham stimulation. Participants were blind to conditions. taVNS was delivered continuously for 5 min and it was composed by trains of 15 pulses (inter-pulse interval of 40 ms; pulse duration = 100 μs), with an inter-train interval of 5 s, namely 60 trains with 900 pulses were given in one session. These settings were derived from previous studies investigating the effects of taVNS in a variety of clinical conditions (Yap et al., 2020). In the present study, we set the stimulus parameters in the range used in most of these studies. Stimulation intensity was adjusted for each subject and defined as a value 1.5 mV above the perception threshold of the electrical stimulation. To this end, participants reported their subjective feelings in a series of short trials of 2 s of stimulation. Stimulation intensity started at 0.1 mA and increased gradually by 0.1 mA in each trial after the participant’s response, until the perception threshold was found. In any case, the intensity was kept below the subjective pain threshold. The mean intensity value was 5.6 mA (range 3.0-8.3 mA).

### 2.4 Statistical analysis

Statistical analysis was performed using R (R Core Team, 2023) and the “GAMLj3” package.

Prior performing the analysis, the whole dataset was inspected for missing data. Two subjects withdrawn from the study after the first arm of the trial (one real stimulation, one sham stimulation). The missing data were deemed as missing completely at random (MCAR). No imputation was performed as linear mixed models can handle missing data using maximum likelihood estimation.

Descriptive statistics were calculated for the demographics and neuropsychological scores of the enrolled subjects (age, years of education, MMSE score, MoCA score).

In order to assess the effect of taVNS over the total number of errors, we used a linear mixed model analysis of variance (ANOVA) with a restricted maximum likelihood (REML) estimation, allowing us to consider both fixed and random effects of multiple independent variables over the dependent variable. The model controlled for within-subject multiple measurements considering the subjects as a clustering factor with a random effect over the intercept. Covariance of residuals was modeled with compound symmetry, as we assumed that all pairs of repeated measures had the same correlation, given the complete randomization of the task. First, we fitted a model which included order of intervention, intervention, timepoint and their interactions as fixed between-subjects factors, as well as the number of days between interventions as a covariate. This model showed no association of the interval length between sessions and the total errors, thus excluding carry over and task learning confounding. Consequently, this factor was dropped from the analysis. We then fitted a model which included demographics and neuropsychological variables (sex, age, years of education, MMSE, and MoCA scores) as well as the span length. Sex and span length and their interactions were included as fixed between-subjects factors; age, years of education, MMSE and MoCA scores were included as covariates. This model showed no significant main effect of sex, meanwhile, as expected, a significant effect was found for span length. As for the covariates, only the MoCA score showed a correlation with the total errors. Therefore, only the span length and MoCA score were kept in the analysis. In the final model, the total errors number was the dependent variable; Order of Intervention, Intervention (Real or Sham), Timepoint (Pre and Post), Span length and the interaction (Intervention*Timepoint*Span_length) were included as fixed between-subjects factors; the MoCA score was included as a covariate. Results were considered statistically significant for *p* < 0.05. Given the design of the study, we were interested in the fixed effect of the Intervention*Timepoint*Span_length interaction to assess the differences between Intervention groups at different Timepoints, for different lengths of the span sequence, controlling for the MoCA score. The Bonferroni method was used for post hoc multiple comparison correction of the Intervention*Timepoint*Span_length interaction.

According to the same principles, two further models were also used for analyzing the secondary outcome measures, considering the pupil size during the encoding and recall periods as the dependent variable. Covariance of residuals was modeled with a first order auto regression model as we assumed that the correlation among repeated measures was expected to be higher for stimulation sessions closer in time and decrease as the time lags increased between sessions. No correlation was found between the MoCA score and pupil size, therefore this variable was dropped from the final model.

## 3 Results

A total of 18 subjects (9 females) were enrolled in the study. Table 1 shows the descriptive statistics of the demographics and neuropsychological assessment of the participants. No adverse event was reported during or after the stimulation protocol.

**TABLE 1:** Descriptive statistics of demographics and neuropsychological tests.

	Age (years)	Education (years)	MMSE	MoCA
Mean	73.5	14.3	27.8	24.4
Standard deviation	4.71	1.71	1.81	3.29
Minimum	66	12	25	17
Maximum	81	16	30	30

### 3.1 Digit span score

**FIGURE 2:**
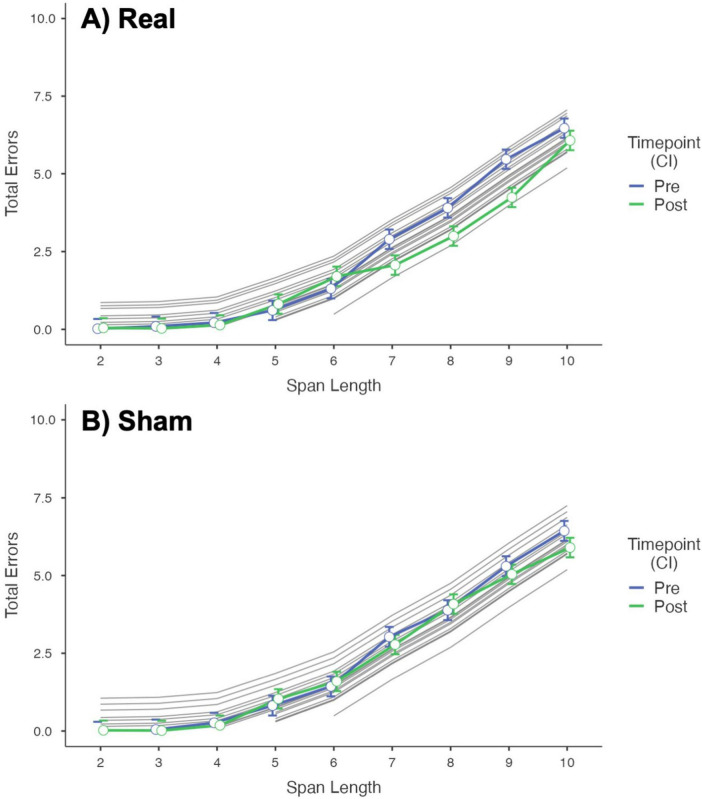
Plot of the fixed effect of timepoint (pre- vs. post-stimulation) over the total errors at different span lengths. **(A)** Results for the Real Intervention showing a significant difference in number of errors after stimulation (fewer errors after stimulation) at span length 7, 8, and 9. **(B)** Results for the Sham Intervention showing no difference in Total Errors before and after stimulation. Random effects are plotted across subjects in gray.

Complete model information is presented in Supplementary material. No significant interaction was found for Order*Intervention*Timepoint. The model shows a significant effect of Intervention*Timepoint*Span_length over the total errors score (*p* < 0.001). A significant inverse correlation was found between the MoCA and total errors scores (i.e., one point increase in MoCA score correlates with a mean decrease of 0.12 in the number of errors, *p* < 0.001). Figure 2 shows a plot of the fixed effect of Timepoint (Pre and Post-stimulation) on Total Errors, at different Span Lengths, for each Intervention group. Post Real stimulation, the mean Total Errors at span length 7, 8, and 9 significantly (after Bonferroni correction) decreased by –0.83 (C.I. –1.15 to –0.50, *p* < 0.001), –0.90 (C.I. –1.23 to –0.58, *p* < 0.001), and –0.39 (C.I. –0.72 to –0.06, *p* < 0.001), respectively, compared to Pre-stimulation values. No difference was found for the Sham Intervention (see Supplementary material).

**FIGURE 3:**
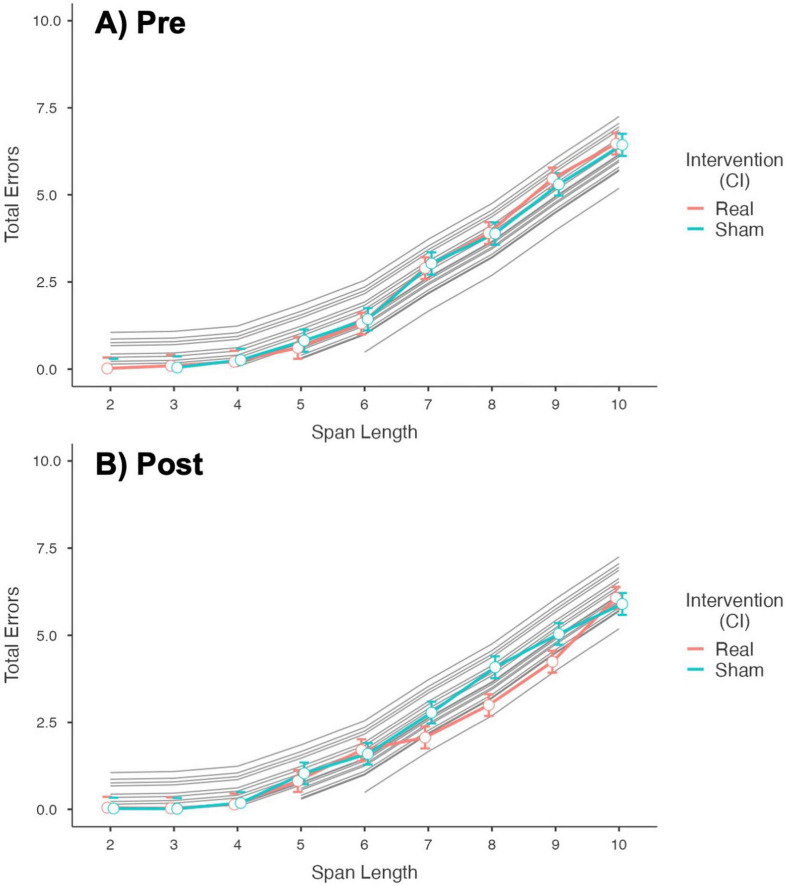
Plot of the fixed effect of intervention (real vs. sham stimulation) over the total errors at different span lengths. **(A)** Results for the Pre-stimulation Timepoint showing no difference between Real and Sham. **(B)** Results for the Post-stimulation Timepoint showing a significant difference in number of errors between Real and Sham (fewer errors for Real) at span length 7, 8, and 9. Random effects are plotted across subjects in gray.

Figure 3 shows a plot of the fixed effect of Intervention (Real and Sham stimulation) on Total Errors, at different Span Lengths, for each Timepoint. No difference was found between Real and Sham in Total Errors Pre-stimulation. Post-stimulation, the mean Total Errors at span length 7, 8, and 9 were significantly (after Bonferroni correction) lower for Real stimulation compared to Sham, of –0.71 (C.I. –0.38 to –1.04, *p* = 0.012), –1.08 (C.I. –0.75 to –1.41, *p* < .001), and –0.79 (C.I. –0.47 to –1.12, *p* = 0.001), respectively (see Supplementary material).

### 3.2 Pupil size

**FIGURE 4:**
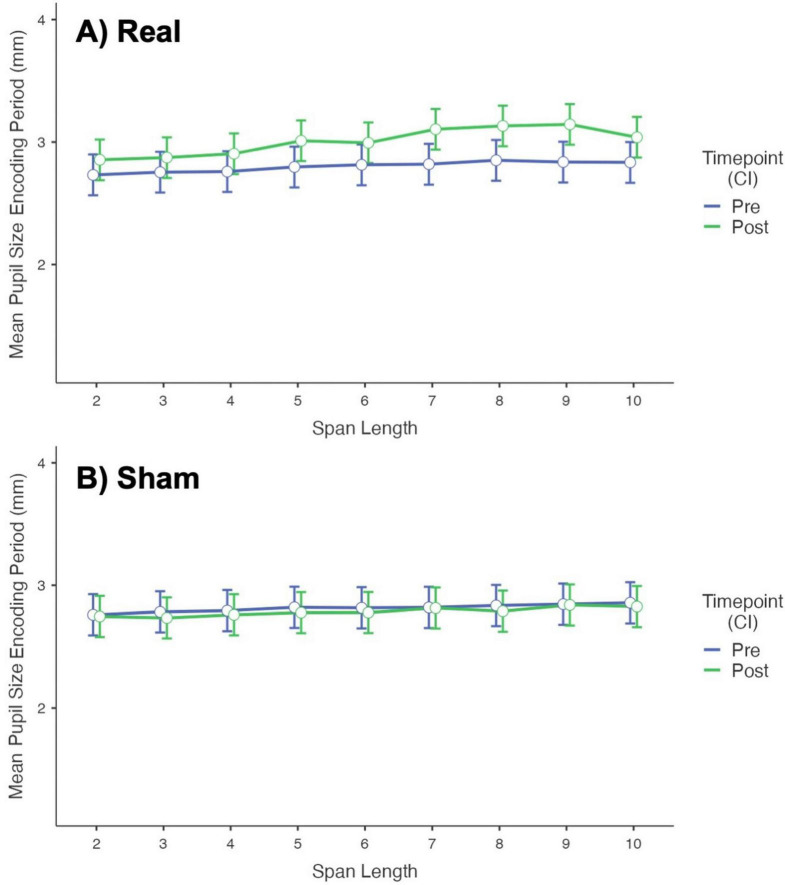
Plot of the fixed effect of timepoint (pre- vs. post-stimulation) over the mean pupil size during the encoding period at different span lengths. **(A)** Results for the Real Intervention showing a significant difference in Mean Pupil Size after stimulation (larger size after stimulation) at all span lengths, with larger effects for span 7-10. **(B)** Results for the Sham Intervention showing no difference in Mean Pupil Size before and after stimulation.

Complete models’ information is presented in Supplementary material. No significant interaction was found for Order*Intervention*Timepoint. For the Encoding Period, the model shows a significant effect of Intervention*Timepoint*Span_Length over the Mean Pupil Size (*p* < 0.001). Figure 4 shows a plot of the fixed effect of Timepoint (Pre and Post-stimulation) on Mean Pupil Size during the Encoding Period, at different Span Lengths, for each Intervention group. Post Real stimulation, the mean pupil size at all span lengths, slightly but significantly (after Bonferroni correction, *p* < 0.001) increased compared to pre-stimulation values, with larger effects for span 7-10. No difference was found for the Sham Intervention (see Supplementary material).

**FIGURE 5:**
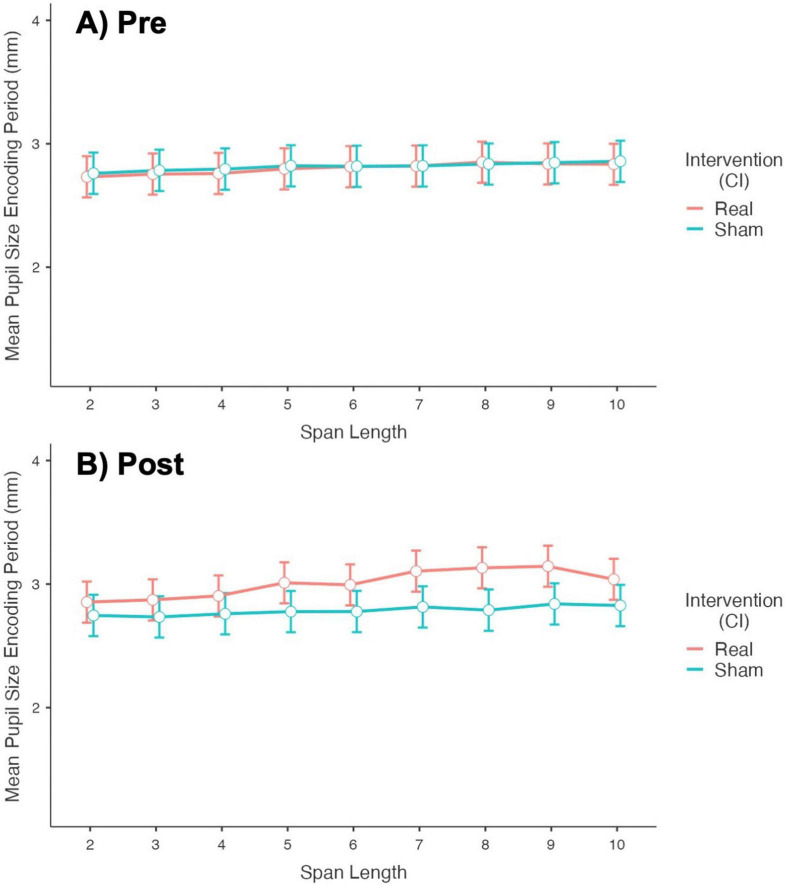
Plot of the fixed effect of intervention (real vs. sham stimulation) over the mean pupil size during the encoding period at different span lengths. **(A)** Results for the Pre-stimulation Timepoint showing no difference between Real and Sham. **(B)** Results for the Post-stimulation Timepoint showing a significant difference in Mean Pupil Size between Real and Sham (larger pupil size for Real) at all span lengths, with larger effects for span 5-10.

Figure 5 shows a plot of the fixed effect of Intervention (Real and Sham stimulation) on Mean Pupil Size during the Encoding Period, at different Span Lengths, for each Timepoint. No difference was found between Real and Sham in Mean Pupil Size at Pre-stimulation phase. In post-stimulation, the Mean Pupil Size at all span lengths were slightly but significantly (after Bonferroni correction, *p* < 0.001) higher for Real stimulation compared to Sham, with larger effects for span 5-10 (see Supplementary material).

No effect of stimulation on Mean Pupil Size was found during the Recall Period (see Supplementary material).

## 4 Discussion

In this pilot study we investigated the effects of a taVNS off-line stimulation paradigm over a digit span task in healthy volunteers. Our results show that, compared to sham stimulation, the real stimulation was able to reduce the total number of errors performed by the subjects. The results were statistically significant for certain span lengths (namely, 7, 8, and 9), with a significant reduction in the number of errors compared to pre-stimulation values. Additionally, the pupil size was measured as an indirect measure of cognitive load. We found that real taVNS increased the pupil size at all span lengths (in particular at 5-10) during the encoding period of the digit span task with no effects during the recall period. The sham stimulation procedure had no effects on the pupil size during either the encoding or recall period.

The digit span task is one of the commonly used indices of STM, correlating with higher-order cognitive functions (Unsworth and Engle, 2007a,b). About 89% of normal subjects has a forward digit span between 5 and 8 (Kaplan, 1991). The average digit span for normal adults without error is 7 ± 2 (Miller, 1956). Accordingly, we found a positive association between the span length and the total number of errors in the digit span task. Given that we were interested in the effect of taVNS at selected sequences, the span length was included in the linear mixed model as a fixed effect term. At span lengths 7, 8, and 9, we found a significant reduction in the mean number of errors following real stimulation compared to the baseline values, whereas no difference was found following sham stimulation. Additionally, a direct comparison between real and sham procedures showed a statistically significant lower number of errors at the same span lengths following real stimulation, while no difference was found for baseline values.

These results suggest that taVNS can improve the STM performance around the usual range of saturation of the forward digit span task (seven plus two). Given the overall low number of errors, at shorter span lengths (below seven) there was no benefit of taVNS. Similarly, at the longest span (ten), no beneficial effect of taVNS was found either. This might be due to a cognitive ceiling effect in the digit span task. Nonetheless, it has been demonstrated that some training strategies might increase the longest digit span recall length (Norris et al., 2019). Therefore, it is possible that different taVNS parameters or stimulation protocols might be effective even for longer span sequences. Future studies are needed to elucidate the better optimal stimulation setting and to further validate this hypothesis.

Previous studies have also shown that the digit span score does not correlate with age (Grégoire and Van Der Linden, 1997) or sex (Lynn and Irwing, 2008). This was the case for the present study as well. On the other hand, the digit span is known to correlate with the education level (Ostrosky-Solís and Lozano, 2006). In our study, we could not find such an effect of education, probably due to the homogenous level of scholarity of our sample.

Regarding cognitive assessment, the MMSE (Folstein et al., 1975) is a general cognitive screening tool which does not include a digit span item. Therefore, no correlation was expected with the number of errors in the digit span task. Meanwhile, the MoCA (Nasreddine et al., 2005) is a more complex cognitive screening tool which includes forward and backward digit span items. It has been shown that MoCA reflects similar constructs as those measured by a more comprehensive battery, including the digit span (Vogel et al., 2015). As such, a correlation between the MoCA score and the score of a digit span task is to be expected. Indeed, we found a significant inverse correlation between MoCA scores and total number of errors. For this reason, in order to correct the total number of errors for the MoCA score, we included it as a covariate in the linear mixed model.

The neurophysiological bases of working memory have been studied extensively with a variety of techniques. Using functional neuroimaging, several brain regions have been implicated in the neural basis of working memory. In particular, a positron emission tomography (PET) study shown that the right dorsolateral prefrontal cortex and bilateral inferior parietal lobule, as well as the anterior cingulate cortex, were activated during digit span tasks. The degree of activation increased with increasing task difficulty in digit forward. Medial occipital cortex, was also activated, suggesting a possible visual imagery strategy for these aural–verbal tasks (Gerton et al., 2004). A fMRI study found that different areas were involved in storing and manipulating processes in verbal working memory (Tsukiura et al., 2001). Additionally, the inferior parietal and inferior frontal cortex were implicated in working memory in subjects with focal brain lesions (Baldo and Dronkers, 2006). This was further confirmed by a study performed on subjects with direct electrical stimulation (Papagno et al., 2017).

On these bases, the selective non-invasive neuromodulation of brain areas involved in these processes might provide an effective way to improve working memory in normal subjects, as well as to provide a therapeutic option in patients with various neurological disorders. In fact, numerous behavioral benefits of cervical VNS likely arise from the vagus nerve’s highly diffuse afferent brain targets. Accordingly, several imaging studies revealed that various cortex and sub-cortex regions highly associated with cognitive performance were regulated by taVNS (Yakunina et al., 2017; Badran et al., 2018).

Overall, there is growing literature suggesting the therapeutic effect of taVNS in a wide range of conditions (Gerges et al., 2024), including cognitive impairment (Wang et al., 2022). Furthermore, previous studies showed that taVNS could significantly improve the cognitive performances of healthy volunteers (Ridgewell et al., 2021), modulating divergent thinking (Colzato et al., 2018), inhibitory control processes (Beste et al., 2016), emotion recognition (Colzato et al., 2017), attentional processes (Ventura-Bort et al., 2018), and working motivation (Neuser et al., 2020). Also, taVNS can effectively ameliorate spatial working memory performance (Sun et al., 2021). A study demonstrated that on-line taVNS was capable to boost associative memory in older individuals, although no effect was found on forward digit span (Jacobs et al., 2015). In the present study, we found that off-line taVNS is capable of improving the performance of healthy elders in a digit forward task. In the field of non-invasive brain stimulation, it is still debated if there is any difference between on-line and off-line protocols in modulation effect of working memory. Meta-analyses of transcranial direct current stimulation (tDCS) studies have suggested that for healthy population the significant effect could be found only in off-line stimulation (Hill et al., 2016), which might be caused by different neurobiological processes. The on-line effects might result from resting membrane potential alterations, whereas the off-line effects might result from modulation of synaptic plasticity (Stagg and Nitsche, 2011; Medeiros et al., 2012; Hill et al., 2016). Similarly to our results, a previous study investigating the effects of taVNS on spatial working memory found that neuromodulation occurred only with off-line stimulation, while no effect was found with on-line stimulation (Sun et al., 2021). The authors proposed that, given that the effect of taVNS on working memory is mediated by the LC-NE release system, it needs time to take effect, which might lead to a stronger effect of off-line protocol than on-line stimulation (Sun et al., 2021). Ludwig et al. (2025) presented participants with emotionally negative or neutral images and applied a brief 3-s taVNS during encoding. During recall, they found that real taVNS selectively enhanced memory for negative stimuli, both immediately and after a 24-h delay, compared to sham or no stimulation. The authors suggested that short bursts of taVNS—whether real or sham—induced pupil dilation and improved memory performance over time, likely through attention-enhancing sensory stimulation. Similarly, in the present study, taVNS may have facilitated digit span performance by enhancing arousal and attentional engagement through LC-NE system activation.

To further assess the effects of taVNS over the cognitive task, we also measured the pupil size as an indirect measure of cognitive load. Previous studies showed that pupil dilation can be used as a measure of task engagement (Johnson et al., 2014). Researchers have consistently shown that adults’ pupils dilate incrementally with each digit encoded in a digit span task until the length of the digit sequence exceeds STM capacity, at which point pupil size begins to plateau or diminish (Cabestrero et al., 2009). Findings from the present study seem to support the prolonged plateau model, showing a progressive slight increase in pupil size across the different span lengths. Regarding the effects of taVNS over pupil size, a previous study showed that taVNS is capable to induce a robust pupil size dilatation (Sharon et al., 2021). This finding was also confirmed by a subsequent replication study (Lloyd et al., 2023), thus supporting the hypothesis that taVNS elevates noradrenaline and other arousal-promoting neuromodulatory signaling, although other studies reported mixed results regarding pupil dilation (Capone et al., 2021; Keute et al., 2019). Philips et al. (2025) showed that high-frequency (300 Hz) taVNS can increase pupil size, but the relationship between stimulus intensity and pupil diameter was non-linear; pupil diameter increased with intensity up to a certain intensity (2-4.8 mA), beyond which the effect plateaued or even reversed. In our study, the stimulation intensity was mostly within the rising and plateau phases of the linear increase in pupil diameter.

In our experiment we found that real taVNS slightly but significantly increased pupil size at all span lengths, with larger effects for longer span lengths, only during the encoding phase of the task. On the other hand, no effect of taVNS on pupil size was found during the recall phase. Previous studies investigating working memory with different experimental paradigms described distinct pupil size differences between the encoding and recall phases of the memory tasks (Kucewicz et al., 2018). In particular, a larger pupil size during encoding was significantly associated with higher likelihood of subsequently recalling a word, presumably reflecting the intensity of attention devoted during encoding. On the other hand, there was no clear evidence of a significant relationship between working memory capacity and subsequent memory recall (Micula et al., 2022).

The present study has some limitations. First, as often occurs in most non-invasive brain stimulation studies, double blinding is not practically achievable. Therefore, participants were blinded to the intervention allocation but the operators who administered taVNS were not. In order to reduce the risk of bias, the cognitive task was scored by an operator blinded to the subject allocation.

The most common disadvantages of cross-over trials are order effect and carry-over between interventions (Lim and In, 2021). To reduce the possibility of an order effect, enrolled subjects were randomized with a 1:1 blocked randomization in the two intervention sequences. In the linear mixed model, the order of intervention was included as a fixed effect factor. We could not find any significant interaction of order with timepoint and interventions, thus excluding any sequence effect.

As for carry-over effect, subjects underwent a wash-out period of at least 1 week before being switched over to the other intervention. Currently, it is not well known how long the effects of taVNS last. According to the literature, repeated administrations of taVNS may lead to long lasting effects, persisting for a few weeks (de Moraes et al., 2023). However, single applications of taVNS do not seem to have any long-term effect (Gurtubay et al., 2023). Consequently, we deemed that, for the current study design, a 1 week wash-out period was enough to avoid any carry-over effect.

A further possible limitation is the possibility of a learning effect experienced by subjects with repeated neuropsychological testing (Tao et al., 2019). In order to prevent any learning, the digit span task in the present study consisted of 63 trials of series of numbers, of different length, which were randomized for each session. We verified these assumptions including in the preliminary linear mixed model the number of days between interventions as a covariate. The model showed no effect of time over the outcome variables, suggesting no carry-over or learning effects were present.

Lastly, findings from this study relate to a population of elderly Japanese. Additional research is needed to establish the generalizability of these results to different demographics and in the context of neurological disorders.

## 5 Conclusion

In this pilot trial, we investigated the effects of a taVNS off-line stimulation paradigm over a digit span task in elderly volunteers. We found that taVNS is capable to improve the short-term memory performance, reducing the total number of errors at selected span lengths. This finding is further supported by the corresponding increase in pupil size found during the encoding phase of the task, indicating that taVNS might be able to selectively modulate attentive processes. No effects were found for sham stimulation, showing that the effects are likely mediated by increased attention and not by a non-specific sensory arousal due to electric stimulation of the skin. Findings from this study could support the use of taVNS as a safe, non-invasive neuromodulation technique to improve cognitive function in both healthy subjects and patients with cognitive impairment, although further studies are needed to clarify the better optimal stimulation parameters.

## Data Availability

The raw data supporting the conclusions of this article will be made available by the authors, without undue reservation.
