# Corrigendum: Effects of off-line auricular transcutaneous vagus nerve stimulation (taVNS) on a short-term memory task: a pilot study

**DOI:** 10.3389/fnagi.2025.1626368

**Published:** 2025-05-26

**Authors:** Francesco Fisicaro, Klizia Cortese, Rita Bella, Manuela Pennisi, Giuseppe Lanza, Kaoru Yuasa, Yoshikazu Ugawa, Yasuo Terao

**Affiliations:** ^1^Department of Biomedical and Biotechnological Sciences, University of Catania, Catania, Italy; ^2^Department of Medical Physiology, Kyorin University, Shinkawa, Tokyo, Japan; ^3^Department of Educational Sciences, University of Catania, Catania, Italy; ^4^Department of Medical and Surgical Sciences and Advanced Technologies, University of Catania, Catania, Italy; ^5^Department of Surgery and Medical-Surgical Specialties, University of Catania, Catania, Italy; ^6^Clinical Neurophysiology Research Unit, Oasi Research Institute-IRCCS, Troina, Italy; ^7^Department of Human Neurophysiology, School of Medicine, Fukushima Medical University, Fukushima, Japan

**Keywords:** transcutaneous auricular vagus nerve stimulation, pupil size, digit span, short term memory, non-invasive brain stimulation

In the published article, there was an error in the Funding statement. Instead of “The author(s) declare that financial support was received for the research and/or publication of this article. FF was supported by a Research Fellowship Grant from the International Federation of Clinical Neurophysiology; YT was supported by a Research Project Grant in-aid for Scientific Research from Communications R&D Promotion Programme from the Ministry of Internal Affairs and Communications, Japan (B203060001),” the corrected Funding statement should have been: “The author(s) declare that financial support was received for the research and/or publication of this article. FF was supported by a Research Fellowship Grant from the International Federation of Clinical Neurophysiology; this work was conducted under the project by Ministry of Internal Affairs and Communications (JPMI10001), Japan.”

The authors apologize for this error and state that this does not change the scientific conclusions of the article in any way. The original article has been updated.

