# Supplementary material for: Effects of off-line auricular transcutaneous vagus nerve stimulation (taVNS) on a short-term memory task: a pilot study
Source: Front Aging Neurosci. 2025 Apr 28;17:1549167. doi: 10.3389/fnagi.2025.1549167 (PMC12066449; doi:10.3389/fnagi.2025.1549167)
Supplement: Supplementary file 1 [file Table_1.docx]

Supplementary Material

# Supplementary Tables

## Model results for digit span score

| Model Info | | | | | |
| --- | --- | --- | --- | --- | --- |
| **Info** | |  | |  | |
| Model Type |  | Mixed Model |  | Linear Mixed model for continuous y |  |
| Model |  | lme |  | `Total Errors` ~ 1 + Intervention + Timepoint + MoCA + `Span Length` + Order + Intervention:Timepoint + Intervention:`Span Length` + Timepoint:`Span Length` + Intervention:Order + Timepoint:Order + Intervention:Timepoint:`Span Length` + Intervention:Timepoint:Order + ( 1 \| Subject ) |  |
| Distribution |  | Gaussian |  | Normal distribution of residuals |  |
| Direction |  | y |  | Dependend variable scores |  |
| Residuals |  | Compound Symmetry |  | within cluster Subject |  |
| Sample size |  | 4221 |  |  |  |
| Converged |  | yes |  |  |  |
| Y transform |  | none |  |  |  |
| C.I. method |  | Wald |  |  |  |
|  | | | | | |

| Model Fit | | | | | | | | | |
| --- | --- | --- | --- | --- | --- | --- | --- | --- | --- |
| **Type** | | **R²** | | **df** | | **LRT X²** | | **p** | |
| Conditional |  | 0.755 |  | 42 |  | 5794.685 |  | < .001 |  |
| Marginal |  | 0.735 |  | 40 |  | 5680.456 |  | < .001 |  |
|  | | | | | | | | | |

| Fixed Effects Omnibus Tests | | | | | | | | | |
| --- | --- | --- | --- | --- | --- | --- | --- | --- | --- |
|  | | **F** | | **df** | | **df (res)** | | **p** | |
| Intervention |  | 13.99 |  | 1 |  | 4165.0 |  | < .001 |  |
| Timepoint |  | 22.72 |  | 1 |  | 4165.0 |  | < .001 |  |
| MoCA |  | 18.65 |  | 1 |  | 15.0 |  | < .001 |  |
| Span Length |  | 1506.31 |  | 8 |  | 4165.0 |  | < .001 |  |
| Order |  | 4.65 |  | 1 |  | 4165.0 |  | 0.031 |  |
| Intervention ✻ Timepoint |  | 10.65 |  | 1 |  | 4165.0 |  | 0.001 |  |
| Intervention ✻ Span Length |  | 3.72 |  | 8 |  | 4165.0 |  | < .001 |  |
| Timepoint ✻ Span Length |  | 8.84 |  | 8 |  | 4165.0 |  | < .001 |  |
| Intervention ✻ Order |  | 1.84 |  | 1 |  | 15.0 |  | 0.195 |  |
| Timepoint ✻ Order |  | 4.16 |  | 1 |  | 4165.0 |  | 0.041 |  |
| Intervention ✻ Timepoint ✻ Span Length |  | 4.35 |  | 8 |  | 4165.0 |  | < .001 |  |
| Intervention ✻ Timepoint ✻ Order |  | 2.70 |  | 1 |  | 4165.0 |  | 0.100 |  |
|  | | | | | | | | | |

| Parameter Estimates (Fixed coefficients) | | | | | | | | | | | | | | | | | | | | | | | | |  |  |
| --- | --- | --- | --- | --- | --- | --- | --- | --- | --- | --- | --- | --- | --- | --- | --- | --- | --- | --- | --- | --- | --- | --- | --- | --- | --- | --- |
|  | | | | | | | | **95% Confidence Intervals** | | | | | | |  | | | | | | | | | |  |  |
| **Names** | | **Effect** | | **Estimate** | | **SE** | | | **Lower** | | | **Upper** | | | | **df** | | | **t** | | | **p** | | | |  |
| (Intercept) |  | (Intercept) |  | 2.27739 |  | 0.0893 |  | | | 2.10232 |  | | 2.45247 |  | | | 4165.0 |  | | 25.5026 |  | | < .001 |  | | |
| Intervention1 |  | Sham - Real |  | 0.15261 |  | 0.0408 |  | | | 0.07262 |  | | 0.23259 |  | | | 4165.0 |  | | 3.7404 |  | | < .001 |  | | |
| Timepoint1 |  | Post - Pre |  | -0.18957 |  | 0.0398 |  | | | -0.26755 |  | | -0.11160 |  | | | 4165.0 |  | | -4.7667 |  | | < .001 |  | | |
| MoCA |  | MoCA |  | -0.12140 |  | 0.0281 |  | | | -0.18131 |  | | -0.06148 |  | | | 15.0 |  | | -4.3187 |  | | < .001 |  | | |
| Span Length1 |  | 3 - 2 |  | 0.03046 |  | 0.0838 |  | | | -0.13391 |  | | 0.19483 |  | | | 4165.0 |  | | 0.3633 |  | | 0.716 |  | | |
| Span Length2 |  | 4 - 2 |  | 0.18277 |  | 0.0838 |  | | | 0.01840 |  | | 0.34715 |  | | | 4165.0 |  | | 2.1800 |  | | 0.029 |  | | |
| Span Length3 |  | 5 - 2 |  | 0.80016 |  | 0.0838 |  | | | 0.63579 |  | | 0.96453 |  | | | 4165.0 |  | | 9.5438 |  | | < .001 |  | | |
| Span Length4 |  | 6 - 2 |  | 1.49199 |  | 0.0838 |  | | | 1.32762 |  | | 1.65636 |  | | | 4165.0 |  | | 17.7955 |  | | < .001 |  | | |
| Span Length5 |  | 7 - 2 |  | 2.67516 |  | 0.0838 |  | | | 2.51079 |  | | 2.83953 |  | | | 4165.0 |  | | 31.9076 |  | | < .001 |  | | |
| Span Length6 |  | 8 - 2 |  | 3.70037 |  | 0.0838 |  | | | 3.53600 |  | | 3.86474 |  | | | 4165.0 |  | | 44.1357 |  | | < .001 |  | | |
| Span Length7 |  | 9 - 2 |  | 4.99422 |  | 0.0838 |  | | | 4.82985 |  | | 5.15860 |  | | | 4165.0 |  | | 59.5680 |  | | < .001 |  | | |
| Span Length8 |  | 10 - 2 |  | 6.19997 |  | 0.0838 |  | | | 6.03560 |  | | 6.36435 |  | | | 4165.0 |  | | 73.9495 |  | | < .001 |  | | |
| Order1 |  | 2 - 1 |  | -0.08807 |  | 0.0408 |  | | | -0.16810 |  | | -0.00804 |  | | | 4165.0 |  | | -2.1575 |  | | 0.031 |  | | |
| Intervention1 ✻ Timepoint1 |  | (Sham - Real) ✻ (Post - Pre) |  | 0.25952 |  | 0.0795 |  | | | 0.10358 |  | | 0.41546 |  | | | 4165.0 |  | | 3.2627 |  | | 0.001 |  | | |
| Intervention1 ✻ Span Length1 |  | (Sham - Real) ✻ (3 - 2) |  | 0.00210 |  | 0.1677 |  | | | -0.32664 |  | | 0.33085 |  | | | 4165.0 |  | | 0.0125 |  | | 0.990 |  | | |
| Intervention1 ✻ Span Length2 |  | (Sham - Real) ✻ (4 - 2) |  | 0.07983 |  | 0.1677 |  | | | -0.24891 |  | | 0.40858 |  | | | 4165.0 |  | | 0.4761 |  | | 0.634 |  | | |
| Intervention1 ✻ Span Length3 |  | (Sham - Real) ✻ (5 - 2) |  | 0.24737 |  | 0.1677 |  | | | -0.08137 |  | | 0.57612 |  | | | 4165.0 |  | | 1.4753 |  | | 0.140 |  | | |
| Intervention1 ✻ Span Length4 |  | (Sham - Real) ✻ (6 - 2) |  | 0.04280 |  | 0.1677 |  | | | -0.28594 |  | | 0.37155 |  | | | 4165.0 |  | | 0.2553 |  | | 0.799 |  | | |
| Intervention1 ✻ Span Length5 |  | (Sham - Real) ✻ (7 - 2) |  | 0.45956 |  | 0.1677 |  | | | 0.13081 |  | | 0.78830 |  | | | 4165.0 |  | | 2.7407 |  | | 0.006 |  | | |
| Intervention1 ✻ Span Length6 |  | (Sham - Real) ✻ (8 - 2) |  | 0.56880 |  | 0.1677 |  | | | 0.24006 |  | | 0.89755 |  | | | 4165.0 |  | | 3.3922 |  | | < .001 |  | | |
| Intervention1 ✻ Span Length7 |  | (Sham - Real) ✻ (9 - 2) |  | 0.34979 |  | 0.1677 |  | | | 0.02104 |  | | 0.67853 |  | | | 4165.0 |  | | 2.0860 |  | | 0.037 |  | | |
| Intervention1 ✻ Span Length8 |  | (Sham - Real) ✻ (10 - 2) |  | -0.07064 |  | 0.1677 |  | | | -0.39939 |  | | 0.25810 |  | | | 4165.0 |  | | -0.4213 |  | | 0.674 |  | | |
| Timepoint1 ✻ Span Length1 |  | (Post - Pre) ✻ (3 - 2) |  | -0.08613 |  | 0.1677 |  | | | -0.41488 |  | | 0.24261 |  | | | 4165.0 |  | | -0.5137 |  | | 0.608 |  | | |
| Timepoint1 ✻ Span Length2 |  | (Post - Pre) ✻ (4 - 2) |  | -0.11345 |  | 0.1677 |  | | | -0.44219 |  | | 0.21530 |  | | | 4165.0 |  | | -0.6766 |  | | 0.499 |  | | |
| Timepoint1 ✻ Span Length3 |  | (Post - Pre) ✻ (5 - 2) |  | 0.17279 |  | 0.1677 |  | | | -0.15595 |  | | 0.50154 |  | | | 4165.0 |  | | 1.0305 |  | | 0.303 |  | | |
| Timepoint1 ✻ Span Length4 |  | (Post - Pre) ✻ (6 - 2) |  | 0.24291 |  | 0.1677 |  | | | -0.08584 |  | | 0.57165 |  | | | 4165.0 |  | | 1.4486 |  | | 0.148 |  | | |
| Timepoint1 ✻ Span Length5 |  | (Post - Pre) ✻ (7 - 2) |  | -0.57721 |  | 0.1677 |  | | | -0.90595 |  | | -0.24846 |  | | | 4165.0 |  | | -3.4423 |  | | < .001 |  | | |
| Timepoint1 ✻ Span Length6 |  | (Post - Pre) ✻ (8 - 2) |  | -0.39233 |  | 0.1677 |  | | | -0.72108 |  | | -0.06359 |  | | | 4165.0 |  | | -2.3397 |  | | 0.019 |  | | |
| Timepoint1 ✻ Span Length7 |  | (Post - Pre) ✻ (9 - 2) |  | -0.77836 |  | 0.1677 |  | | | -1.10711 |  | | -0.44962 |  | | | 4165.0 |  | | -4.6419 |  | | < .001 |  | | |
| Timepoint1 ✻ Span Length8 |  | (Post - Pre) ✻ (10 - 2) |  | -0.50079 |  | 0.1677 |  | | | -0.82953 |  | | -0.17204 |  | | | 4165.0 |  | | -2.9865 |  | | 0.003 |  | | |
| Intervention1 ✻ Order1 |  | (Sham - Real) ✻ (2 - 1) |  | -0.48707 |  | 0.3591 |  | | | -1.25247 |  | | 0.27834 |  | | | 15.0 |  | | -1.3564 |  | | 0.195 |  | | |
| Timepoint1 ✻ Order1 |  | (Post - Pre) ✻ (2 - 1) |  | 0.16230 |  | 0.0795 |  | | | 0.00635 |  | | 0.31824 |  | | | 4165.0 |  | | 2.0404 |  | | 0.041 |  | | |
| Intervention1 ✻ Timepoint1 ✻ Span Length1 |  | (Sham - Real) ✻ (Post - Pre) ✻ (3 - 2) |  | 0.01261 |  | 0.3354 |  | | | -0.64489 |  | | 0.67010 |  | | | 4165.0 |  | | 0.0376 |  | | 0.970 |  | | |
| Intervention1 ✻ Timepoint1 ✻ Span Length2 |  | (Sham - Real) ✻ (Post - Pre) ✻ (4 - 2) |  | -0.02521 |  | 0.3354 |  | | | -0.68270 |  | | 0.63228 |  | | | 4165.0 |  | | -0.0752 |  | | 0.940 |  | | |
| Intervention1 ✻ Timepoint1 ✻ Span Length3 |  | (Sham - Real) ✻ (Post - Pre) ✻ (5 - 2) |  | -0.00735 |  | 0.3354 |  | | | -0.66484 |  | | 0.65014 |  | | | 4165.0 |  | | -0.0219 |  | | 0.983 |  | | |
| Intervention1 ✻ Timepoint1 ✻ Span Length4 |  | (Sham - Real) ✻ (Post - Pre) ✻ (6 - 2) |  | -0.25368 |  | 0.3354 |  | | | -0.91117 |  | | 0.40381 |  | | | 4165.0 |  | | -0.7564 |  | | 0.449 |  | | |
| Intervention1 ✻ Timepoint1 ✻ Span Length5 |  | (Sham - Real) ✻ (Post - Pre) ✻ (7 - 2) |  | 0.55987 |  | 0.3354 |  | | | -0.09762 |  | | 1.21736 |  | | | 4165.0 |  | | 1.6695 |  | | 0.095 |  | | |
| Intervention1 ✻ Timepoint1 ✻ Span Length6 |  | (Sham - Real) ✻ (Post - Pre) ✻ (8 - 2) |  | 1.08088 |  | 0.3354 |  | | | 0.42339 |  | | 1.73837 |  | | | 4165.0 |  | | 3.2230 |  | | 0.001 |  | | |
| Intervention1 ✻ Timepoint1 ✻ Span Length7 |  | (Sham - Real) ✻ (Post - Pre) ✻ (9 - 2) |  | 0.94748 |  | 0.3354 |  | | | 0.28999 |  | | 1.60497 |  | | | 4165.0 |  | | 2.8252 |  | | 0.005 |  | | |
| Intervention1 ✻ Timepoint1 ✻ Span Length8 |  | (Sham - Real) ✻ (Post - Pre) ✻ (10 - 2) |  | -0.16124 |  | 0.3354 |  | | | -0.81873 |  | | 0.49625 |  | | | 4165.0 |  | | -0.4808 |  | | 0.631 |  | | |
| Intervention1 ✻ Timepoint1 ✻ Order1 |  | (Sham - Real) ✻ (Post - Pre) ✻ (2 - 1) |  | 0.26154 |  | 0.1591 |  | | | -0.05034 |  | | 0.57343 |  | | | 4165.0 |  | | 1.6441 |  | | 0.100 |  | | |
|  | | | | | | | | | | | | | | | | | | | | | | | | |  |  |

| Random Components | | | | | | | | | | | |
| --- | --- | --- | --- | --- | --- | --- | --- | --- | --- | --- | --- |
| **Groups** | | **Name** | | **Variance** | | **SD** | | **ICC** | | **rho** | |
| Subject |  | (Intercept) |  | 0.136 |  | 0.369 |  | 0.0763 |  | 1.73e-18 |  |
| Residual |  |  |  | 1.647 |  | 1.283 |  |  |  |  |  |
| Note. Number of Obs: 4221, Number of groups: Subject 18 | | | | | | | | | | | |
|  | | | | | | | | | | | |

### Simple Effects

| ANOVA for Simple Effects of Timepoint | | | | | | | | | | | |
| --- | --- | --- | --- | --- | --- | --- | --- | --- | --- | --- | --- |
| **Moderator** | | | |  | | | | | | | |
| **Intervention** | | **Span Length** | | **F** | | **Num df** | | **Den df** | | **p** | |
| Real |  | 2 |  | 0.0250 |  | 1.00 |  | 4165 |  | 0.875 |  |
|  |  | 3 |  | 0.1590 |  | 1.00 |  | 4165 |  | 0.690 |  |
|  |  | 4 |  | 0.2010 |  | 1.00 |  | 4165 |  | 0.654 |  |
|  |  | 5 |  | 1.4820 |  | 1.00 |  | 4165 |  | 0.223 |  |
|  |  | 6 |  | 5.6590 |  | 1.00 |  | 4165 |  | 0.017 |  |
|  |  | 7 |  | 24.9350 |  | 1.00 |  | 4165 |  | < .001 |  |
|  |  | 8 |  | 29.6800 |  | 1.00 |  | 4165 |  | < .001 |  |
|  |  | 9 |  | 54.2690 |  | 1.00 |  | 4165 |  | < .001 |  |
|  |  | 10 |  | 5.6060 |  | 1.00 |  | 4165 |  | 0.018 |  |
| Sham |  | 2 |  | 0.0750 |  | 1.00 |  | 4165 |  | 0.784 |  |
|  |  | 3 |  | 0.0390 |  | 1.00 |  | 4165 |  | 0.843 |  |
|  |  | 4 |  | 0.2220 |  | 1.00 |  | 4165 |  | 0.638 |  |
|  |  | 5 |  | 1.6230 |  | 1.00 |  | 4165 |  | 0.203 |  |
|  |  | 6 |  | 0.9220 |  | 1.00 |  | 4165 |  | 0.337 |  |
|  |  | 7 |  | 2.1990 |  | 1.00 |  | 4165 |  | 0.138 |  |
|  |  | 8 |  | 1.3220 |  | 1.00 |  | 4165 |  | 0.250 |  |
|  |  | 9 |  | 2.3300 |  | 1.00 |  | 4165 |  | 0.127 |  |
|  |  | 10 |  | 10.0020 |  | 1.00 |  | 4165 |  | 0.002 |  |
|  | | | | | | | | | | | |

| Parameter Estimates for simple effects of Timepoint | | | | | | | | | | | | | | | | | | | |
| --- | --- | --- | --- | --- | --- | --- | --- | --- | --- | --- | --- | --- | --- | --- | --- | --- | --- | --- | --- |
| **Moderator** | | | |  | | | | | | **95% Confidence Intervals** | | | |  | | | | | |
| **Intervention** | | **Span Length** | | **Effect** | | **Estimate** | | **SE** | | **Lower** | | **Upper** | | **df** | | **t** | | **p** | |
| Real |  | 2 |  | Post - Pre |  | 0.0261 |  | 0.166 |  | -0.3001 |  | 0.3524 |  | 4165 |  | 0.157 |  | 0.875 |  |
|  |  | 3 |  | Post - Pre |  | -0.0663 |  | 0.166 |  | -0.3926 |  | 0.2600 |  | 4165 |  | -0.398 |  | 0.690 |  |
|  |  | 4 |  | Post - Pre |  | -0.0747 |  | 0.166 |  | -0.4010 |  | 0.2516 |  | 4165 |  | -0.449 |  | 0.654 |  |
|  |  | 5 |  | Post - Pre |  | 0.2026 |  | 0.166 |  | -0.1237 |  | 0.5289 |  | 4165 |  | 1.217 |  | 0.223 |  |
|  |  | 6 |  | Post - Pre |  | 0.3959 |  | 0.166 |  | 0.0696 |  | 0.7222 |  | 4165 |  | 2.379 |  | 0.017 |  |
|  |  | 7 |  | Post - Pre |  | -0.8310 |  | 0.166 |  | -1.1573 |  | -0.5047 |  | 4165 |  | -4.993 |  | < .001 |  |
|  |  | 8 |  | Post - Pre |  | -0.9066 |  | 0.166 |  | -1.2329 |  | -0.5804 |  | 4165 |  | -5.448 |  | < .001 |  |
|  |  | 9 |  | Post - Pre |  | -1.2260 |  | 0.166 |  | -1.5522 |  | -0.8997 |  | 4165 |  | -7.367 |  | < .001 |  |
|  |  | 10 |  | Post - Pre |  | -0.3940 |  | 0.166 |  | -0.7203 |  | -0.0678 |  | 4165 |  | -2.368 |  | 0.018 |  |
| Sham |  | 2 |  | Post - Pre |  | 0.0464 |  | 0.169 |  | -0.2853 |  | 0.3781 |  | 4165 |  | 0.274 |  | 0.784 |  |
|  |  | 3 |  | Post - Pre |  | -0.0334 |  | 0.169 |  | -0.3651 |  | 0.2982 |  | 4165 |  | -0.198 |  | 0.843 |  |
|  |  | 4 |  | Post - Pre |  | -0.0797 |  | 0.169 |  | -0.4113 |  | 0.2520 |  | 4165 |  | -0.471 |  | 0.638 |  |
|  |  | 5 |  | Post - Pre |  | 0.2155 |  | 0.169 |  | -0.1161 |  | 0.5472 |  | 4165 |  | 1.274 |  | 0.203 |  |
|  |  | 6 |  | Post - Pre |  | 0.1625 |  | 0.169 |  | -0.1692 |  | 0.4941 |  | 4165 |  | 0.960 |  | 0.337 |  |
|  |  | 7 |  | Post - Pre |  | -0.2509 |  | 0.169 |  | -0.5825 |  | 0.0808 |  | 4165 |  | -1.483 |  | 0.138 |  |
|  |  | 8 |  | Post - Pre |  | 0.1945 |  | 0.169 |  | -0.1372 |  | 0.5262 |  | 4165 |  | 1.150 |  | 0.250 |  |
|  |  | 9 |  | Post - Pre |  | -0.2582 |  | 0.169 |  | -0.5899 |  | 0.0734 |  | 4165 |  | -1.526 |  | 0.127 |  |
|  |  | 10 |  | Post - Pre |  | -0.5350 |  | 0.169 |  | -0.8667 |  | -0.2034 |  | 4165 |  | -3.163 |  | 0.002 |  |
|  | | | | | | | | | | | | | | | | | | | |

| ANOVA for Simple Effects of Intervention | | | | | | | | | | | |
| --- | --- | --- | --- | --- | --- | --- | --- | --- | --- | --- | --- |
| **Moderator** | | | |  | | | | | | | |
| **Timepoint** | | **Span Length** | | **F** | | **Num df** | | **Den df** | | **p** | |
| Pre |  | 2 |  | 0.06800 |  | 1.00 |  | 4165 |  | 0.794 |  |
|  |  | 3 |  | 0.08100 |  | 1.00 |  | 4165 |  | 0.775 |  |
|  |  | 4 |  | 0.08100 |  | 1.00 |  | 4165 |  | 0.776 |  |
|  |  | 5 |  | 1.49200 |  | 1.00 |  | 4165 |  | 0.222 |  |
|  |  | 6 |  | 0.54900 |  | 1.00 |  | 4165 |  | 0.459 |  |
|  |  | 7 |  | 0.63900 |  | 1.00 |  | 4165 |  | 0.424 |  |
|  |  | 8 |  | 0.00900 |  | 1.00 |  | 4165 |  | 0.926 |  |
|  |  | 9 |  | 0.98500 |  | 1.00 |  | 4165 |  | 0.321 |  |
|  |  | 10 |  | 0.04100 |  | 1.00 |  | 4165 |  | 0.840 |  |
| Post |  | 2 |  | 0.02100 |  | 1.00 |  | 4165 |  | 0.886 |  |
|  |  | 3 |  | 0.00900 |  | 1.00 |  | 4165 |  | 0.926 |  |
|  |  | 4 |  | 0.06800 |  | 1.00 |  | 4165 |  | 0.795 |  |
|  |  | 5 |  | 1.73900 |  | 1.00 |  | 4165 |  | 0.187 |  |
|  |  | 6 |  | 0.41900 |  | 1.00 |  | 4165 |  | 0.517 |  |
|  |  | 7 |  | 18.43500 |  | 1.00 |  | 4165 |  | < .001 |  |
|  |  | 8 |  | 42.40700 |  | 1.00 |  | 4165 |  | < .001 |  |
|  |  | 9 |  | 23.01900 |  | 1.00 |  | 4165 |  | < .001 |  |
|  |  | 10 |  | 1.10400 |  | 1.00 |  | 4165 |  | 0.293 |  |
|  | | | | | | | | | | | |

| Parameter Estimates for simple effects of Intervention | | | | | | | | | | | | | | | | | | | |
| --- | --- | --- | --- | --- | --- | --- | --- | --- | --- | --- | --- | --- | --- | --- | --- | --- | --- | --- | --- |
| **Moderator** | | | |  | | | | | | **95% Confidence Intervals** | | | |  | | | | | |
| **Timepoint** | | **Span Length** | | **Effect** | | **Estimate** | | **SE** | | **Lower** | | **Upper** | | **df** | | **t** | | **p** | |
| Pre |  | 2 |  | Sham - Real |  | -0.0441 |  | 0.169 |  | -0.376 |  | 0.288 |  | 4165 |  | -0.2606 |  | 0.794 |  |
|  |  | 3 |  | Sham - Real |  | -0.0483 |  | 0.169 |  | -0.380 |  | 0.284 |  | 4165 |  | -0.2854 |  | 0.775 |  |
|  |  | 4 |  | Sham - Real |  | 0.0483 |  | 0.169 |  | -0.284 |  | 0.380 |  | 4165 |  | 0.2850 |  | 0.776 |  |
|  |  | 5 |  | Sham - Real |  | 0.2069 |  | 0.169 |  | -0.125 |  | 0.539 |  | 4165 |  | 1.2213 |  | 0.222 |  |
|  |  | 6 |  | Sham - Real |  | 0.1255 |  | 0.169 |  | -0.207 |  | 0.458 |  | 4165 |  | 0.7408 |  | 0.459 |  |
|  |  | 7 |  | Sham - Real |  | 0.1355 |  | 0.169 |  | -0.197 |  | 0.468 |  | 4165 |  | 0.7997 |  | 0.424 |  |
|  |  | 8 |  | Sham - Real |  | -0.0158 |  | 0.169 |  | -0.348 |  | 0.316 |  | 4165 |  | -0.0932 |  | 0.926 |  |
|  |  | 9 |  | Sham - Real |  | -0.1681 |  | 0.169 |  | -0.500 |  | 0.164 |  | 4165 |  | -0.9922 |  | 0.321 |  |
|  |  | 10 |  | Sham - Real |  | -0.0342 |  | 0.169 |  | -0.366 |  | 0.298 |  | 4165 |  | -0.2017 |  | 0.840 |  |
| Post |  | 2 |  | Sham - Real |  | -0.0239 |  | 0.167 |  | -0.351 |  | 0.303 |  | 4165 |  | -0.1433 |  | 0.886 |  |
|  |  | 3 |  | Sham - Real |  | -0.0155 |  | 0.167 |  | -0.342 |  | 0.311 |  | 4165 |  | -0.0929 |  | 0.926 |  |
|  |  | 4 |  | Sham - Real |  | 0.0433 |  | 0.167 |  | -0.283 |  | 0.370 |  | 4165 |  | 0.2600 |  | 0.795 |  |
|  |  | 5 |  | Sham - Real |  | 0.2198 |  | 0.167 |  | -0.107 |  | 0.547 |  | 4165 |  | 1.3188 |  | 0.187 |  |
|  |  | 6 |  | Sham - Real |  | -0.1079 |  | 0.167 |  | -0.435 |  | 0.219 |  | 4165 |  | -0.6475 |  | 0.517 |  |
|  |  | 7 |  | Sham - Real |  | 0.7156 |  | 0.167 |  | 0.389 |  | 1.042 |  | 4165 |  | 4.2936 |  | < .001 |  |
|  |  | 8 |  | Sham - Real |  | 1.0854 |  | 0.167 |  | 0.759 |  | 1.412 |  | 4165 |  | 6.5121 |  | < .001 |  |
|  |  | 9 |  | Sham - Real |  | 0.7996 |  | 0.167 |  | 0.473 |  | 1.126 |  | 4165 |  | 4.7978 |  | < .001 |  |
|  |  | 10 |  | Sham - Real |  | -0.1752 |  | 0.167 |  | -0.502 |  | 0.152 |  | 4165 |  | -1.0509 |  | 0.293 |  |
|  | | | | | | | | | | | | | | | | | | | |

## Model results for pupil size – encoding

| Model Info | | | | | |
| --- | --- | --- | --- | --- | --- |
| **Info** | |  | |  | |
| Model Type |  | Mixed Model |  | Linear Mixed model for continuous y |  |
| Model |  | lme |  | `Mean Pupil Size Encoding Period (mm)` ~ 1 + Intervention + Timepoint + Order + `Span Length` + Intervention:Timepoint + Intervention:Order + Timepoint:Order + Intervention:`Span Length` + Timepoint:`Span Length` + Intervention:Timepoint:Order + Intervention:Timepoint:`Span Length` + ( 1 \| Subject ) |  |
| Distribution |  | Gaussian |  | Normal distribution of residuals |  |
| Direction |  | y |  | Dependend variable scores |  |
| Residuals |  | Autoregressive 1 |  | within cluster Subject |  |
| Sample size |  | 4158 |  |  |  |
| Converged |  | yes |  |  |  |
| Y transform |  | none |  |  |  |
| C.I. method |  | Wald |  |  |  |
|  | | | | | |

| Model Fit | | | | | | | | | |
| --- | --- | --- | --- | --- | --- | --- | --- | --- | --- |
| **Type** | | **R²** | | **df** | | **LRT X²** | | **p** | |
| Conditional |  | 0.719 |  | 41 |  | 9605.251 |  | < .001 |  |
| Marginal |  | 0.216 |  | 39 |  | 1171.005 |  | < .001 |  |
|  | | | | | | | | | |

| Fixed Effects Omnibus Tests | | | | | | | | | |
| --- | --- | --- | --- | --- | --- | --- | --- | --- | --- |
|  | | **F** | | **df** | | **df (res)** | | **p** | |
| Intervention |  | 34.075 |  | 1 |  | 4102.0 |  | < .001 |  |
| Timepoint |  | 19.262 |  | 1 |  | 4102.0 |  | < .001 |  |
| Order |  | 54.172 |  | 1 |  | 4102.0 |  | < .001 |  |
| Span Length |  | 126.577 |  | 8 |  | 4102.0 |  | < .001 |  |
| Intervention ✻ Timepoint |  | 49.003 |  | 1 |  | 4102.0 |  | < .001 |  |
| Intervention ✻ Order |  | 4.822 |  | 1 |  | 16.0 |  | 0.043 |  |
| Timepoint ✻ Order |  | 0.301 |  | 1 |  | 4102.0 |  | 0.583 |  |
| Intervention ✻ Span Length |  | 25.866 |  | 8 |  | 4102.0 |  | < .001 |  |
| Timepoint ✻ Span Length |  | 18.575 |  | 8 |  | 4102.0 |  | < .001 |  |
| Intervention ✻ Timepoint ✻ Order |  | 1.556 |  | 1 |  | 4102.0 |  | 0.212 |  |
| Intervention ✻ Timepoint ✻ Span Length |  | 12.849 |  | 8 |  | 4102.0 |  | < .001 |  |
|  | | | | | | | | | |

| Parameter Estimates (Fixed coefficients) | | | | | | | | | | | | | | | | | |
| --- | --- | --- | --- | --- | --- | --- | --- | --- | --- | --- | --- | --- | --- | --- | --- | --- | --- |
|  | | | | | | | | **95% Confidence Intervals** | | | |  | | | | | |
| **Names** | | **Effect** | | **Estimate** | | **SE** | | **Lower** | | **Upper** | | **df** | | **t** | | **p** | |
| (Intercept) |  | (Intercept) |  | 2.85132 |  | 0.07695 |  | 2.70047 |  | 3.00218 |  | 4102.0 |  | 37.0559 |  | < .001 |  |
| Intervention1 |  | Sham - Real |  | -0.10247 |  | 0.01755 |  | -0.13688 |  | -0.06805 |  | 4102.0 |  | -5.8373 |  | < .001 |  |
| Timepoint1 |  | Post - Pre |  | 0.08874 |  | 0.02022 |  | 0.04910 |  | 0.12839 |  | 4102.0 |  | 4.3888 |  | < .001 |  |
| Order1 |  | 2 - 1 |  | 0.12904 |  | 0.01753 |  | 0.09467 |  | 0.16342 |  | 4102.0 |  | 7.3602 |  | < .001 |  |
| Span Length1 |  | 3 - 2 |  | 0.01313 |  | 0.00666 |  | 6.76e-5 |  | 0.02619 |  | 4102.0 |  | 1.9707 |  | 0.049 |  |
| Span Length2 |  | 4 - 2 |  | 0.03129 |  | 0.00670 |  | 0.01815 |  | 0.04442 |  | 4102.0 |  | 4.6699 |  | < .001 |  |
| Span Length3 |  | 5 - 2 |  | 0.07828 |  | 0.00667 |  | 0.06521 |  | 0.09136 |  | 4102.0 |  | 11.7352 |  | < .001 |  |
| Span Length4 |  | 6 - 2 |  | 0.07774 |  | 0.00665 |  | 0.06471 |  | 0.09078 |  | 4102.0 |  | 11.6945 |  | < .001 |  |
| Span Length5 |  | 7 - 2 |  | 0.11681 |  | 0.00664 |  | 0.10380 |  | 0.12983 |  | 4102.0 |  | 17.5927 |  | < .001 |  |
| Span Length6 |  | 8 - 2 |  | 0.12881 |  | 0.00666 |  | 0.11575 |  | 0.14186 |  | 4102.0 |  | 19.3474 |  | < .001 |  |
| Span Length7 |  | 9 - 2 |  | 0.14376 |  | 0.00666 |  | 0.13071 |  | 0.15681 |  | 4102.0 |  | 21.5973 |  | < .001 |  |
| Span Length8 |  | 10 - 2 |  | 0.11632 |  | 0.00671 |  | 0.10316 |  | 0.12948 |  | 4102.0 |  | 17.3291 |  | < .001 |  |
| Intervention1 ✻ Timepoint1 |  | (Sham - Real) ✻ (Post - Pre) |  | -0.23674 |  | 0.03382 |  | -0.30304 |  | -0.17043 |  | 4102.0 |  | -7.0002 |  | < .001 |  |
| Intervention1 ✻ Order1 |  | (Sham - Real) ✻ (2 - 1) |  | -0.67583 |  | 0.30779 |  | -1.32831 |  | -0.02336 |  | 16.0 |  | -2.1958 |  | 0.043 |  |
| Timepoint1 ✻ Order1 |  | (Post - Pre) ✻ (2 - 1) |  | -0.01854 |  | 0.03378 |  | -0.08476 |  | 0.04768 |  | 4102.0 |  | -0.5490 |  | 0.583 |  |
| Intervention1 ✻ Span Length1 |  | (Sham - Real) ✻ (3 - 2) |  | -0.01420 |  | 0.01333 |  | -0.04033 |  | 0.01192 |  | 4102.0 |  | -1.0657 |  | 0.287 |  |
| Intervention1 ✻ Span Length2 |  | (Sham - Real) ✻ (4 - 2) |  | -0.01451 |  | 0.01341 |  | -0.04081 |  | 0.01178 |  | 4102.0 |  | -1.0821 |  | 0.279 |  |
| Intervention1 ✻ Span Length3 |  | (Sham - Real) ✻ (5 - 2) |  | -0.06441 |  | 0.01333 |  | -0.09054 |  | -0.03827 |  | 4102.0 |  | -4.8315 |  | < .001 |  |
| Intervention1 ✻ Span Length4 |  | (Sham - Real) ✻ (6 - 2) |  | -0.06648 |  | 0.01327 |  | -0.09250 |  | -0.04046 |  | 4102.0 |  | -5.0088 |  | < .001 |  |
| Intervention1 ✻ Span Length5 |  | (Sham - Real) ✻ (7 - 2) |  | -0.10429 |  | 0.01328 |  | -0.13032 |  | -0.07825 |  | 4102.0 |  | -7.8541 |  | < .001 |  |
| Intervention1 ✻ Span Length6 |  | (Sham - Real) ✻ (8 - 2) |  | -0.13888 |  | 0.01331 |  | -0.16498 |  | -0.11277 |  | 4102.0 |  | -10.4303 |  | < .001 |  |
| Intervention1 ✻ Span Length7 |  | (Sham - Real) ✻ (9 - 2) |  | -0.10757 |  | 0.01331 |  | -0.13367 |  | -0.08147 |  | 4102.0 |  | -8.0808 |  | < .001 |  |
| Intervention1 ✻ Span Length8 |  | (Sham - Real) ✻ (10 - 2) |  | -0.05429 |  | 0.01341 |  | -0.08058 |  | -0.02800 |  | 4102.0 |  | -4.0483 |  | < .001 |  |
| Timepoint1 ✻ Span Length1 |  | (Post - Pre) ✻ (3 - 2) |  | -0.01985 |  | 0.01332 |  | -0.04597 |  | 0.00627 |  | 4102.0 |  | -1.4897 |  | 0.136 |  |
| Timepoint1 ✻ Span Length2 |  | (Post - Pre) ✻ (4 - 2) |  | 0.00111 |  | 0.01340 |  | -0.02518 |  | 0.02739 |  | 4102.0 |  | 0.0825 |  | 0.934 |  |
| Timepoint1 ✻ Span Length3 |  | (Post - Pre) ✻ (5 - 2) |  | 0.03087 |  | 0.01333 |  | 0.00474 |  | 0.05701 |  | 4102.0 |  | 2.3159 |  | 0.021 |  |
| Timepoint1 ✻ Span Length4 |  | (Post - Pre) ✻ (6 - 2) |  | 0.01586 |  | 0.01329 |  | -0.01019 |  | 0.04191 |  | 4102.0 |  | 1.1939 |  | 0.233 |  |
| Timepoint1 ✻ Span Length5 |  | (Post - Pre) ✻ (7 - 2) |  | 0.08591 |  | 0.01329 |  | 0.05985 |  | 0.11198 |  | 4102.0 |  | 6.4628 |  | < .001 |  |
| Timepoint1 ✻ Span Length6 |  | (Post - Pre) ✻ (8 - 2) |  | 0.06298 |  | 0.01331 |  | 0.03688 |  | 0.08907 |  | 4102.0 |  | 4.7309 |  | < .001 |  |
| Timepoint1 ✻ Span Length7 |  | (Post - Pre) ✻ (9 - 2) |  | 0.09609 |  | 0.01330 |  | 0.07001 |  | 0.12217 |  | 4102.0 |  | 7.2239 |  | < .001 |  |
| Timepoint1 ✻ Span Length8 |  | (Post - Pre) ✻ (10 - 2) |  | 0.03239 |  | 0.01342 |  | 0.00608 |  | 0.05871 |  | 4102.0 |  | 2.4132 |  | 0.016 |  |
| Intervention1 ✻ Timepoint1 ✻ Order1 |  | (Sham - Real) ✻ (Post - Pre) ✻ (2 - 1) |  | 0.10088 |  | 0.08087 |  | -0.05766 |  | 0.25942 |  | 4102.0 |  | 1.2475 |  | 0.212 |  |
| Intervention1 ✻ Timepoint1 ✻ Span Length1 |  | (Sham - Real) ✻ (Post - Pre) ✻ (3 - 2) |  | -0.03139 |  | 0.02665 |  | -0.08364 |  | 0.02086 |  | 4102.0 |  | -1.1777 |  | 0.239 |  |
| Intervention1 ✻ Timepoint1 ✻ Span Length2 |  | (Sham - Real) ✻ (Post - Pre) ✻ (4 - 2) |  | -0.04337 |  | 0.02677 |  | -0.09585 |  | 0.00912 |  | 4102.0 |  | -1.6199 |  | 0.105 |  |
| Intervention1 ✻ Timepoint1 ✻ Span Length3 |  | (Sham - Real) ✻ (Post - Pre) ✻ (5 - 2) |  | -0.12127 |  | 0.02669 |  | -0.17359 |  | -0.06895 |  | 4102.0 |  | -4.5444 |  | < .001 |  |
| Intervention1 ✻ Timepoint1 ✻ Span Length4 |  | (Sham - Real) ✻ (Post - Pre) ✻ (6 - 2) |  | -0.08227 |  | 0.02658 |  | -0.13438 |  | -0.03016 |  | 4102.0 |  | -3.0954 |  | 0.002 |  |
| Intervention1 ✻ Timepoint1 ✻ Span Length5 |  | (Sham - Real) ✻ (Post - Pre) ✻ (7 - 2) |  | -0.15445 |  | 0.02658 |  | -0.20657 |  | -0.10234 |  | 4102.0 |  | -5.8102 |  | < .001 |  |
| Intervention1 ✻ Timepoint1 ✻ Span Length6 |  | (Sham - Real) ✻ (Post - Pre) ✻ (8 - 2) |  | -0.19066 |  | 0.02663 |  | -0.24287 |  | -0.13844 |  | 4102.0 |  | -7.1586 |  | < .001 |  |
| Intervention1 ✻ Timepoint1 ✻ Span Length7 |  | (Sham - Real) ✻ (Post - Pre) ✻ (9 - 2) |  | -0.17810 |  | 0.02660 |  | -0.23026 |  | -0.12594 |  | 4102.0 |  | -6.6948 |  | < .001 |  |
| Intervention1 ✻ Timepoint1 ✻ Span Length8 |  | (Sham - Real) ✻ (Post - Pre) ✻ (10 - 2) |  | -0.09966 |  | 0.02683 |  | -0.15226 |  | -0.04706 |  | 4102.0 |  | -3.7144 |  | < .001 |  |
|  | | | | | | | | | | | | | | | | | |

| Random Components | | | | | | | | | | | |
| --- | --- | --- | --- | --- | --- | --- | --- | --- | --- | --- | --- |
| **Groups** | | **Name** | | **Variance** | | **SD** | | **ICC** | | **Phi** | |
| Subject |  | (Intercept) |  | 0.1037 |  | 0.322 |  | 0.641 |  | 0.837 |  |
| Residual |  |  |  | 0.0580 |  | 0.241 |  |  |  |  |  |
| Note. Number of Obs: 4158, Number of groups: Subject 18 | | | | | | | | | | | |
|  | | | | | | | | | | | |

### Simple Effects

| ANOVA for Simple Effects of Timepoint | | | | | | | | | | | |
| --- | --- | --- | --- | --- | --- | --- | --- | --- | --- | --- | --- |
| **Moderator** | | | |  | | | | | | | |
| **Intervention** | | **Span Length** | | **F** | | **Num df** | | **Den df** | | **p** | |
| Real |  | 2 |  | 19.5410 |  | 1.00 |  | 4102 |  | < .001 |  |
|  |  | 3 |  | 18.5160 |  | 1.00 |  | 4102 |  | < .001 |  |
|  |  | 4 |  | 28.2700 |  | 1.00 |  | 4102 |  | < .001 |  |
|  |  | 5 |  | 60.3510 |  | 1.00 |  | 4102 |  | < .001 |  |
|  |  | 6 |  | 43.8210 |  | 1.00 |  | 4102 |  | < .001 |  |
|  |  | 7 |  | 107.2460 |  | 1.00 |  | 4102 |  | < .001 |  |
|  |  | 8 |  | 103.7670 |  | 1.00 |  | 4102 |  | < .001 |  |
|  |  | 9 |  | 126.3450 |  | 1.00 |  | 4102 |  | < .001 |  |
|  |  | 10 |  | 55.3000 |  | 1.00 |  | 4102 |  | < .001 |  |
| Sham |  | 2 |  | 0.1900 |  | 1.00 |  | 4102 |  | 0.663 |  |
|  |  | 3 |  | 2.5610 |  | 1.00 |  | 4102 |  | 0.110 |  |
|  |  | 4 |  | 1.2190 |  | 1.00 |  | 4102 |  | 0.270 |  |
|  |  | 5 |  | 2.0090 |  | 1.00 |  | 4102 |  | 0.156 |  |
|  |  | 6 |  | 1.6070 |  | 1.00 |  | 4102 |  | 0.205 |  |
|  |  | 7 |  | 0.0240 |  | 1.00 |  | 4102 |  | 0.876 |  |
|  |  | 8 |  | 2.2610 |  | 1.00 |  | 4102 |  | 0.133 |  |
|  |  | 9 |  | 0.0440 |  | 1.00 |  | 4102 |  | 0.834 |  |
|  |  | 10 |  | 1.0180 |  | 1.00 |  | 4102 |  | 0.313 |  |
|  | | | | | | | | | | | |

| Parameter Estimates for simple effects of Timepoint | | | | | | | | | | | | | | | | | | | |
| --- | --- | --- | --- | --- | --- | --- | --- | --- | --- | --- | --- | --- | --- | --- | --- | --- | --- | --- | --- |
| **Moderator** | | | |  | | | | | | **95% Confidence Intervals** | | | |  | | | | | |
| **Intervention** | | **Span Length** | | **Effect** | | **Estimate** | | **SE** | | **Lower** | | **Upper** | | **df** | | **t** | | **p** | |
| Real |  | 2 |  | Post - Pre |  | 0.12312 |  | 0.0279 |  | 0.0685 |  | 0.1777 |  | 4102 |  | 4.421 |  | < .001 |  |
|  |  | 3 |  | Post - Pre |  | 0.11896 |  | 0.0276 |  | 0.0648 |  | 0.1732 |  | 4102 |  | 4.303 |  | < .001 |  |
|  |  | 4 |  | Post - Pre |  | 0.14591 |  | 0.0274 |  | 0.0921 |  | 0.1997 |  | 4102 |  | 5.317 |  | < .001 |  |
|  |  | 5 |  | Post - Pre |  | 0.21463 |  | 0.0276 |  | 0.1605 |  | 0.2688 |  | 4102 |  | 7.769 |  | < .001 |  |
|  |  | 6 |  | Post - Pre |  | 0.18012 |  | 0.0272 |  | 0.1268 |  | 0.2335 |  | 4102 |  | 6.620 |  | < .001 |  |
|  |  | 7 |  | Post - Pre |  | 0.28626 |  | 0.0276 |  | 0.2321 |  | 0.3405 |  | 4102 |  | 10.356 |  | < .001 |  |
|  |  | 8 |  | Post - Pre |  | 0.28142 |  | 0.0276 |  | 0.2273 |  | 0.3356 |  | 4102 |  | 10.187 |  | < .001 |  |
|  |  | 9 |  | Post - Pre |  | 0.30826 |  | 0.0274 |  | 0.2545 |  | 0.3620 |  | 4102 |  | 11.240 |  | < .001 |  |
|  |  | 10 |  | Post - Pre |  | 0.20534 |  | 0.0276 |  | 0.1512 |  | 0.2595 |  | 4102 |  | 7.436 |  | < .001 |  |
| Sham |  | 2 |  | Post - Pre |  | -0.01349 |  | 0.0310 |  | -0.0742 |  | 0.0472 |  | 4102 |  | -0.436 |  | 0.663 |  |
|  |  | 3 |  | Post - Pre |  | -0.04903 |  | 0.0306 |  | -0.1091 |  | 0.0110 |  | 4102 |  | -1.600 |  | 0.110 |  |
|  |  | 4 |  | Post - Pre |  | -0.03407 |  | 0.0309 |  | -0.0946 |  | 0.0264 |  | 4102 |  | -1.104 |  | 0.270 |  |
|  |  | 5 |  | Post - Pre |  | -0.04325 |  | 0.0305 |  | -0.1031 |  | 0.0166 |  | 4102 |  | -1.417 |  | 0.156 |  |
|  |  | 6 |  | Post - Pre |  | -0.03876 |  | 0.0306 |  | -0.0987 |  | 0.0212 |  | 4102 |  | -1.268 |  | 0.205 |  |
|  |  | 7 |  | Post - Pre |  | -0.00480 |  | 0.0307 |  | -0.0650 |  | 0.0554 |  | 4102 |  | -0.156 |  | 0.876 |  |
|  |  | 8 |  | Post - Pre |  | -0.04584 |  | 0.0305 |  | -0.1056 |  | 0.0139 |  | 4102 |  | -1.504 |  | 0.133 |  |
|  |  | 9 |  | Post - Pre |  | -0.00644 |  | 0.0308 |  | -0.0668 |  | 0.0539 |  | 4102 |  | -0.209 |  | 0.834 |  |
|  |  | 10 |  | Post - Pre |  | -0.03092 |  | 0.0306 |  | -0.0910 |  | 0.0292 |  | 4102 |  | -1.009 |  | 0.313 |  |
|  | | | | | | | | | | | | | | | | | | | |

| ANOVA for Simple Effects of Intervention | | | | | | | | | | | |
| --- | --- | --- | --- | --- | --- | --- | --- | --- | --- | --- | --- |
| **Moderator** | | | |  | | | | | | | |
| **Timepoint** | | **Span Length** | | **F** | | **Num df** | | **Den df** | | **p** | |
| Pre |  | 2 |  | 1.01300 |  | 1.00 |  | 4102 |  | 0.314 |  |
|  |  | 3 |  | 1.16100 |  | 1.00 |  | 4102 |  | 0.281 |  |
|  |  | 4 |  | 1.60500 |  | 1.00 |  | 4102 |  | 0.205 |  |
|  |  | 5 |  | 0.78400 |  | 1.00 |  | 4102 |  | 0.376 |  |
|  |  | 6 |  | 0.01400 |  | 1.00 |  | 4102 |  | 0.907 |  |
|  |  | 7 |  | 0.00300 |  | 1.00 |  | 4102 |  | 0.957 |  |
|  |  | 8 |  | 0.29400 |  | 1.00 |  | 4102 |  | 0.588 |  |
|  |  | 9 |  | 0.13000 |  | 1.00 |  | 4102 |  | 0.719 |  |
|  |  | 10 |  | 0.76100 |  | 1.00 |  | 4102 |  | 0.383 |  |
| Post |  | 2 |  | 15.92600 |  | 1.00 |  | 4102 |  | < .001 |  |
|  |  | 3 |  | 26.90400 |  | 1.00 |  | 4102 |  | < .001 |  |
|  |  | 4 |  | 28.79500 |  | 1.00 |  | 4102 |  | < .001 |  |
|  |  | 5 |  | 74.99600 |  | 1.00 |  | 4102 |  | < .001 |  |
|  |  | 6 |  | 65.92400 |  | 1.00 |  | 4102 |  | < .001 |  |
|  |  | 7 |  | 116.67100 |  | 1.00 |  | 4102 |  | < .001 |  |
|  |  | 8 |  | 161.13000 |  | 1.00 |  | 4102 |  | < .001 |  |
|  |  | 9 |  | 128.61600 |  | 1.00 |  | 4102 |  | < .001 |  |
|  |  | 10 |  | 62.78600 |  | 1.00 |  | 4102 |  | < .001 |  |
|  | | | | | | | | | | | |

| Parameter Estimates for simple effects of Intervention | | | | | | | | | | | | | | | | | | | |
| --- | --- | --- | --- | --- | --- | --- | --- | --- | --- | --- | --- | --- | --- | --- | --- | --- | --- | --- | --- |
| **Moderator** | | | |  | | | | | | **95% Confidence Intervals** | | | |  | | | | | |
| **Timepoint** | | **Span Length** | | **Effect** | | **Estimate** | | **SE** | | **Lower** | | **Upper** | | **df** | | **t** | | **p** | |
| Pre |  | 2 |  | Sham - Real |  | 0.02857 |  | 0.0284 |  | -0.0271 |  | 0.0842 |  | 4102 |  | 1.0063 |  | 0.314 |  |
|  |  | 3 |  | Sham - Real |  | 0.03007 |  | 0.0279 |  | -0.0246 |  | 0.0848 |  | 4102 |  | 1.0774 |  | 0.281 |  |
|  |  | 4 |  | Sham - Real |  | 0.03574 |  | 0.0282 |  | -0.0196 |  | 0.0911 |  | 4102 |  | 1.2668 |  | 0.205 |  |
|  |  | 5 |  | Sham - Real |  | 0.02480 |  | 0.0280 |  | -0.0301 |  | 0.0797 |  | 4102 |  | 0.8857 |  | 0.376 |  |
|  |  | 6 |  | Sham - Real |  | 0.00323 |  | 0.0277 |  | -0.0510 |  | 0.0574 |  | 4102 |  | 0.1167 |  | 0.907 |  |
|  |  | 7 |  | Sham - Real |  | 0.00151 |  | 0.0281 |  | -0.0535 |  | 0.0565 |  | 4102 |  | 0.0538 |  | 0.957 |  |
|  |  | 8 |  | Sham - Real |  | -0.01498 |  | 0.0276 |  | -0.0691 |  | 0.0392 |  | 4102 |  | -0.5423 |  | 0.588 |  |
|  |  | 9 |  | Sham - Real |  | 0.01005 |  | 0.0279 |  | -0.0447 |  | 0.0648 |  | 4102 |  | 0.3602 |  | 0.719 |  |
|  |  | 10 |  | Sham - Real |  | 0.02411 |  | 0.0276 |  | -0.0301 |  | 0.0783 |  | 4102 |  | 0.8726 |  | 0.383 |  |
| Post |  | 2 |  | Sham - Real |  | -0.10804 |  | 0.0271 |  | -0.1611 |  | -0.0550 |  | 4102 |  | -3.9907 |  | < .001 |  |
|  |  | 3 |  | Sham - Real |  | -0.13793 |  | 0.0266 |  | -0.1901 |  | -0.0858 |  | 4102 |  | -5.1869 |  | < .001 |  |
|  |  | 4 |  | Sham - Real |  | -0.14423 |  | 0.0269 |  | -0.1969 |  | -0.0915 |  | 4102 |  | -5.3661 |  | < .001 |  |
|  |  | 5 |  | Sham - Real |  | -0.23308 |  | 0.0269 |  | -0.2858 |  | -0.1803 |  | 4102 |  | -8.6600 |  | < .001 |  |
|  |  | 6 |  | Sham - Real |  | -0.21565 |  | 0.0266 |  | -0.2677 |  | -0.1636 |  | 4102 |  | -8.1194 |  | < .001 |  |
|  |  | 7 |  | Sham - Real |  | -0.28955 |  | 0.0268 |  | -0.3421 |  | -0.2370 |  | 4102 |  | -10.8014 |  | < .001 |  |
|  |  | 8 |  | Sham - Real |  | -0.34224 |  | 0.0270 |  | -0.3951 |  | -0.2894 |  | 4102 |  | -12.6937 |  | < .001 |  |
|  |  | 9 |  | Sham - Real |  | -0.30466 |  | 0.0269 |  | -0.3573 |  | -0.2520 |  | 4102 |  | -11.3409 |  | < .001 |  |
|  |  | 10 |  | Sham - Real |  | -0.21216 |  | 0.0268 |  | -0.2646 |  | -0.1597 |  | 4102 |  | -7.9238 |  | < .001 |  |
|  | | | | | | | | | | | | | | | | | | | |

## Model results for pupil size – recall

| Model Info | | | | | |
| --- | --- | --- | --- | --- | --- |
| **Info** | |  | |  | |
| Model Type |  | Mixed Model |  | Linear Mixed model for continuous y |  |
| Model |  | lme |  | `Mean Pupil Size Recall Period (mm)` ~ 1 + Intervention + Timepoint + Order + `Span Length` + Intervention:Timepoint + Intervention:Order + Timepoint:Order + Intervention:`Span Length` + Timepoint:`Span Length` + Intervention:Timepoint:Order + Intervention:Timepoint:`Span Length` + ( 1 \| Subject ) |  |
| Distribution |  | Gaussian |  | Normal distribution of residuals |  |
| Direction |  | y |  | Dependend variable scores |  |
| Residuals |  | Autoregressive 1 |  | within cluster Subject |  |
| Sample size |  | 4157 |  |  |  |
| Converged |  | yes |  |  |  |
| Y transform |  | none |  |  |  |
| C.I. method |  | Wald |  |  |  |
|  | | | | | |

| Model Fit | | | | | | | | | |
| --- | --- | --- | --- | --- | --- | --- | --- | --- | --- |
| **Type** | | **R²** | | **df** | | **LRT X²** | | **p** | |
| Conditional |  | 0.664 |  | 41 |  | 9628.019 |  | < .001 |  |
| Marginal |  | 0.188 |  | 39 |  | 575.740 |  | < .001 |  |
|  | | | | | | | | | |

| Fixed Effects Omnibus Tests | | | | | | | | | |
| --- | --- | --- | --- | --- | --- | --- | --- | --- | --- |
|  | | **F** | | **df** | | **df (res)** | | **p** | |
| Intervention |  | 4.50616 |  | 1 |  | 4101.0 |  | 0.034 |  |
| Timepoint |  | 7.47731 |  | 1 |  | 4101.0 |  | 0.006 |  |
| Order |  | 40.29857 |  | 1 |  | 4101.0 |  | < .001 |  |
| Span Length |  | 101.87148 |  | 8 |  | 4101.0 |  | < .001 |  |
| Intervention ✻ Timepoint |  | 1.18969 |  | 1 |  | 4101.0 |  | 0.275 |  |
| Intervention ✻ Order |  | 5.40511 |  | 1 |  | 16.0 |  | 0.034 |  |
| Timepoint ✻ Order |  | 0.16986 |  | 1 |  | 4101.0 |  | 0.680 |  |
| Intervention ✻ Span Length |  | 1.10081 |  | 8 |  | 4101.0 |  | 0.359 |  |
| Timepoint ✻ Span Length |  | 0.96338 |  | 8 |  | 4101.0 |  | 0.463 |  |
| Intervention ✻ Timepoint ✻ Order |  | 0.00113 |  | 1 |  | 4101.0 |  | 0.973 |  |
| Intervention ✻ Timepoint ✻ Span Length |  | 0.99969 |  | 8 |  | 4101.0 |  | 0.434 |  |
|  | | | | | | | | | |

| Parameter Estimates (Fixed coefficients) | | | | | | | | | | | | | | | | | |
| --- | --- | --- | --- | --- | --- | --- | --- | --- | --- | --- | --- | --- | --- | --- | --- | --- | --- |
|  | | | | | | | | **95% Confidence Intervals** | | | |  | | | | | |
| **Names** | | **Effect** | | **Estimate** | | **SE** | | **Lower** | | **Upper** | | **df** | | **t** | | **p** | |
| (Intercept) |  | (Intercept) |  | 2.81106 |  | 0.07770 |  | 2.65873 |  | 2.96339 |  | 4101.0 |  | 36.1792 |  | < .001 |  |
| Intervention1 |  | Sham - Real |  | 0.04116 |  | 0.01939 |  | 0.00315 |  | 0.07917 |  | 4101.0 |  | 2.1228 |  | 0.034 |  |
| Timepoint1 |  | Post - Pre |  | -0.06383 |  | 0.02334 |  | -0.10960 |  | -0.01807 |  | 4101.0 |  | -2.7345 |  | 0.006 |  |
| Order1 |  | 2 - 1 |  | 0.12289 |  | 0.01936 |  | 0.08494 |  | 0.16085 |  | 4101.0 |  | 6.3481 |  | < .001 |  |
| Span Length1 |  | 3 - 2 |  | 0.02870 |  | 0.00670 |  | 0.01556 |  | 0.04184 |  | 4101.0 |  | 4.2829 |  | < .001 |  |
| Span Length2 |  | 4 - 2 |  | 0.05362 |  | 0.00674 |  | 0.04041 |  | 0.06684 |  | 4101.0 |  | 7.9566 |  | < .001 |  |
| Span Length3 |  | 5 - 2 |  | 0.10578 |  | 0.00671 |  | 0.09263 |  | 0.11894 |  | 4101.0 |  | 15.7649 |  | < .001 |  |
| Span Length4 |  | 6 - 2 |  | 0.12701 |  | 0.00669 |  | 0.11390 |  | 0.14012 |  | 4101.0 |  | 18.9943 |  | < .001 |  |
| Span Length5 |  | 7 - 2 |  | 0.13466 |  | 0.00668 |  | 0.12157 |  | 0.14776 |  | 4101.0 |  | 20.1622 |  | < .001 |  |
| Span Length6 |  | 8 - 2 |  | 0.11508 |  | 0.00670 |  | 0.10195 |  | 0.12822 |  | 4101.0 |  | 17.1755 |  | < .001 |  |
| Span Length7 |  | 9 - 2 |  | 0.11172 |  | 0.00670 |  | 0.09859 |  | 0.12484 |  | 4101.0 |  | 16.6854 |  | < .001 |  |
| Span Length8 |  | 10 - 2 |  | 0.10937 |  | 0.00675 |  | 0.09613 |  | 0.12261 |  | 4101.0 |  | 16.1966 |  | < .001 |  |
| Intervention1 ✻ Timepoint1 |  | (Sham - Real) ✻ (Post - Pre) |  | 0.04066 |  | 0.03728 |  | -0.03243 |  | 0.11375 |  | 4101.0 |  | 1.0907 |  | 0.275 |  |
| Intervention1 ✻ Order1 |  | (Sham - Real) ✻ (2 - 1) |  | -0.72256 |  | 0.31079 |  | -1.38141 |  | -0.06371 |  | 16.0 |  | -2.3249 |  | 0.034 |  |
| Timepoint1 ✻ Order1 |  | (Post - Pre) ✻ (2 - 1) |  | -0.01534 |  | 0.03722 |  | -0.08832 |  | 0.05764 |  | 4101.0 |  | -0.4121 |  | 0.680 |  |
| Intervention1 ✻ Span Length1 |  | (Sham - Real) ✻ (3 - 2) |  | -0.01427 |  | 0.01340 |  | -0.04055 |  | 0.01201 |  | 4101.0 |  | -1.0649 |  | 0.287 |  |
| Intervention1 ✻ Span Length2 |  | (Sham - Real) ✻ (4 - 2) |  | -0.02309 |  | 0.01349 |  | -0.04954 |  | 0.00336 |  | 4101.0 |  | -1.7113 |  | 0.087 |  |
| Intervention1 ✻ Span Length3 |  | (Sham - Real) ✻ (5 - 2) |  | -0.00822 |  | 0.01341 |  | -0.03451 |  | 0.01807 |  | 4101.0 |  | -0.6130 |  | 0.540 |  |
| Intervention1 ✻ Span Length4 |  | (Sham - Real) ✻ (6 - 2) |  | 0.00126 |  | 0.01335 |  | -0.02491 |  | 0.02744 |  | 4101.0 |  | 0.0946 |  | 0.925 |  |
| Intervention1 ✻ Span Length5 |  | (Sham - Real) ✻ (7 - 2) |  | -0.01705 |  | 0.01336 |  | -0.04323 |  | 0.00914 |  | 4101.0 |  | -1.2763 |  | 0.202 |  |
| Intervention1 ✻ Span Length6 |  | (Sham - Real) ✻ (8 - 2) |  | -0.01101 |  | 0.01340 |  | -0.03728 |  | 0.01526 |  | 4101.0 |  | -0.8215 |  | 0.411 |  |
| Intervention1 ✻ Span Length7 |  | (Sham - Real) ✻ (9 - 2) |  | -0.02785 |  | 0.01339 |  | -0.05411 |  | -0.00160 |  | 4101.0 |  | -2.0802 |  | 0.038 |  |
| Intervention1 ✻ Span Length8 |  | (Sham - Real) ✻ (10 - 2) |  | -0.00551 |  | 0.01349 |  | -0.03196 |  | 0.02094 |  | 4101.0 |  | -0.4082 |  | 0.683 |  |
| Timepoint1 ✻ Span Length1 |  | (Post - Pre) ✻ (3 - 2) |  | 0.00813 |  | 0.01340 |  | -0.01814 |  | 0.03441 |  | 4101.0 |  | 0.6069 |  | 0.544 |  |
| Timepoint1 ✻ Span Length2 |  | (Post - Pre) ✻ (4 - 2) |  | 0.00505 |  | 0.01348 |  | -0.02139 |  | 0.03149 |  | 4101.0 |  | 0.3745 |  | 0.708 |  |
| Timepoint1 ✻ Span Length3 |  | (Post - Pre) ✻ (5 - 2) |  | 0.01359 |  | 0.01341 |  | -0.01270 |  | 0.03988 |  | 4101.0 |  | 1.0134 |  | 0.311 |  |
| Timepoint1 ✻ Span Length4 |  | (Post - Pre) ✻ (6 - 2) |  | -0.00926 |  | 0.01337 |  | -0.03546 |  | 0.01695 |  | 4101.0 |  | -0.6925 |  | 0.489 |  |
| Timepoint1 ✻ Span Length5 |  | (Post - Pre) ✻ (7 - 2) |  | 0.01877 |  | 0.01337 |  | -0.00745 |  | 0.04498 |  | 4101.0 |  | 1.4035 |  | 0.161 |  |
| Timepoint1 ✻ Span Length6 |  | (Post - Pre) ✻ (8 - 2) |  | 0.01704 |  | 0.01340 |  | -0.00923 |  | 0.04330 |  | 4101.0 |  | 1.2718 |  | 0.204 |  |
| Timepoint1 ✻ Span Length7 |  | (Post - Pre) ✻ (9 - 2) |  | 0.01659 |  | 0.01338 |  | -0.00964 |  | 0.04283 |  | 4101.0 |  | 1.2402 |  | 0.215 |  |
| Timepoint1 ✻ Span Length8 |  | (Post - Pre) ✻ (10 - 2) |  | 0.00992 |  | 0.01350 |  | -0.01655 |  | 0.03640 |  | 4101.0 |  | 0.7347 |  | 0.463 |  |
| Intervention1 ✻ Timepoint1 ✻ Order1 |  | (Sham - Real) ✻ (Post - Pre) ✻ (2 - 1) |  | -0.00314 |  | 0.09336 |  | -0.18617 |  | 0.17989 |  | 4101.0 |  | -0.0336 |  | 0.973 |  |
| Intervention1 ✻ Timepoint1 ✻ Span Length1 |  | (Sham - Real) ✻ (Post - Pre) ✻ (3 - 2) |  | 0.04042 |  | 0.02681 |  | -0.01214 |  | 0.09299 |  | 4101.0 |  | 1.5078 |  | 0.132 |  |
| Intervention1 ✻ Timepoint1 ✻ Span Length2 |  | (Sham - Real) ✻ (Post - Pre) ✻ (4 - 2) |  | 0.02209 |  | 0.02693 |  | -0.03070 |  | 0.07489 |  | 4101.0 |  | 0.8204 |  | 0.412 |  |
| Intervention1 ✻ Timepoint1 ✻ Span Length3 |  | (Sham - Real) ✻ (Post - Pre) ✻ (5 - 2) |  | 0.02116 |  | 0.02684 |  | -0.03147 |  | 0.07379 |  | 4101.0 |  | 0.7883 |  | 0.431 |  |
| Intervention1 ✻ Timepoint1 ✻ Span Length4 |  | (Sham - Real) ✻ (Post - Pre) ✻ (6 - 2) |  | 0.04875 |  | 0.02674 |  | -0.00367 |  | 0.10116 |  | 4101.0 |  | 1.8233 |  | 0.068 |  |
| Intervention1 ✻ Timepoint1 ✻ Span Length5 |  | (Sham - Real) ✻ (Post - Pre) ✻ (7 - 2) |  | 0.02267 |  | 0.02674 |  | -0.02975 |  | 0.07510 |  | 4101.0 |  | 0.8480 |  | 0.396 |  |
| Intervention1 ✻ Timepoint1 ✻ Span Length6 |  | (Sham - Real) ✻ (Post - Pre) ✻ (8 - 2) |  | 0.06048 |  | 0.02681 |  | 0.00793 |  | 0.11304 |  | 4101.0 |  | 2.2564 |  | 0.024 |  |
| Intervention1 ✻ Timepoint1 ✻ Span Length7 |  | (Sham - Real) ✻ (Post - Pre) ✻ (9 - 2) |  | 0.02673 |  | 0.02676 |  | -0.02573 |  | 0.07919 |  | 4101.0 |  | 0.9989 |  | 0.318 |  |
| Intervention1 ✻ Timepoint1 ✻ Span Length8 |  | (Sham - Real) ✻ (Post - Pre) ✻ (10 - 2) |  | 0.05081 |  | 0.02699 |  | -0.00212 |  | 0.10373 |  | 4101.0 |  | 1.8822 |  | 0.060 |  |
|  | | | | | | | | | | | | | | | | | |

| Random Components | | | | | | | | | | | |
| --- | --- | --- | --- | --- | --- | --- | --- | --- | --- | --- | --- |
| **Groups** | | **Name** | | **Variance** | | **SD** | | **ICC** | | **Phi** | |
| Subject |  | (Intercept) |  | 0.1042 |  | 0.323 |  | 0.587 |  | 0.868 |  |
| Residual |  |  |  | 0.0734 |  | 0.271 |  |  |  |  |  |
| Note. Number of Obs: 4157, Number of groups: Subject 18 | | | | | | | | | | | |
